# Prevalence and genetic diversity of *Batrachochytrium dendrobatidis* in Central African island and continental amphibian communities

**DOI:** 10.1002/ece3.3309

**Published:** 2017-08-22

**Authors:** Marina E. Hydeman, Ana V. Longo, Guillermo Velo‐Antón, David Rodriguez, Kelly R. Zamudio, Rayna C. Bell

**Affiliations:** ^1^ Department of Ecology and Evolutionary Biology Cornell University Ithaca NY USA; ^2^ CIBIO‐InBIO Universidade do Porto Campus Agrário de Vairão Vairão Portugal; ^3^ Department of Biology Texas State University San Marcos TX USA; ^4^ Department of Vertebrate Zoology National Museum of Natural History Smithsonian Institution Washington DC USA

**Keywords:** *Bd*GPL, caecilian, dilution effect, Equatorial Guinea, Gabon, São Tomé and Príncipe

## Abstract

The fungal pathogen *Batrachochytrium dendrobatidis* (*Bd*) infects hundreds of amphibian species and is implicated in global amphibian declines. *Bd* is comprised of several lineages that differ in pathogenicity, thus, identifying which *Bd* strains are present in a given amphibian community is essential for understanding host–pathogen dynamics. The presence of *Bd* has been confirmed in Central Africa, yet vast expanses of this region have not yet been surveyed for *Bd* prevalence, and the genetic diversity of *Bd* is largely unknown in this part of the world. Using retrospective surveys of museum specimens and contemporary field surveys, we estimated the prevalence of *Bd* in Central African island and continental amphibian assemblages, and genotyped strains of *Bd* present in each community. Our sampling of museum specimens included just a few individuals collected in the Gulf of Guinea archipelago prior to 1998, yet one of these individuals was *Bd*‐positive indicating that the pathogen has been on Bioko Island since 1966. We detected *Bd* across all subsequent sample years in our study and found modest support for a relationship between host life history and *Bd* prevalence, a positive relationship between prevalence and host community species richness, and no significant relationship between elevation and prevalence. The Global Panzootic Lineage (*Bd* GPL) was present in all the island and continental amphibian communities we surveyed. Our results are consistent with a long‐term and widespread distribution of *Bd* in amphibian communities of Gabon and the Gulf of Guinea archipelago.

## 1 INTRODUCTION

*Batrachochytrium dendrobatidis* (*Bd*) is a generalist amphibian pathogen that infects hundreds of species and is implicated in global amphibian declines (Berger et al., 1998; Crawford, Lips, Bermingham, & Wake, 2010; Lips et al., 2006; Olson et al., 2013). Although *Bd* has a widespread distribution, variation in host immune responses (McMahon et al., 2014; Savage & Zamudio, 2011), community structure (Becker & Zamudio, 2011; Becker et al., 2014), environmental conditions (Becker, Rodríguez, Longo, Talaba, & Zamudio, 2012; Longo, Burrowes, & Joglar, 2010), and in the pathogen itself (Farrer et al., 2011) result in variable disease outcomes across the globe. Field surveys of *Bd* typically focus on amphibian host diversity; however, *Bd* is comprised of several lineages that vary in global distribution and in pathogenicity. The most widely distributed and virulent lineage, the Global Panzootic Lineage (*Bd*GPL; Farrer et al., 2011), spread rapidly into new regions and is associated with amphibian declines due to chytridiomycosis in both temperate and tropical amphibian communities (Crawford et al., 2010; Lips, Diffendorfer, Mendelson, & Sears, 2008; Lips et al., 2006; Skerratt et al., 2007; Vredenburg, Knapp, Tunstall, & Briggs, 2010). Other distinct lineages identified to date include strains endemic to South Africa (*Bd*Cape; Farrer et al., 2011), Switzerland (*Bd*CH; Farrer et al., 2011), Brazil (*Bd*Brazil; Schloegel et al., 2012), and Korea (*Bd*Korea; Bataille et al., 2013). Although these other lineages appear to be more geographically restricted, increased geographic and genomic sampling indicates that the biogeography of *Bd* is extremely complex (Bataille et al., 2013; Rodriguez, Becker, Pupin, Haddad, & Zamudio, 2014; Rosenblum et al., 2013), possibly due in part to the global amphibian trade. For example, captive African clawed frogs are the suspected source of *Bd*Cape in endemic midwife toads on the Mediterranean island of Mallorca (Farrer et al., 2011; Walker et al., 2008), and the bullfrog trade may be responsible for introducing *Bd*Brazil into wild amphibian populations in South Korea (Bataille et al., 2013). *Bd* diversity in Africa is largely unexplored beyond South Africa, and no studies have yet linked amphibian population declines to chytridiomycosis; thus, although *Bd* is documented in amphibian communities in West Africa, Central Africa, East Africa, and Madagascar (Bletz et al., 2015; Olson et al., 2013), identifying which strains of *Bd* are present will be essential for understanding future disease dynamics in these communities.

Vast expanses of the African continent have yet to be surveyed for *Bd*; however, the pathogen appears to have a widespread historical presence in Africa (Soto‐Azat, Clarke, Poynton, & Cunningham, 2010). With the exception of Upper Guinean rain forests, west of the Dahomey Gap in West Africa (Penner et al., 2013) and the Seychelles (Labisko et al., 2015), the contemporary distribution of *Bd* encompasses a range of environments and amphibian hosts across the African continent (Doherty‐Bone et al., 2013; Kielgast, Rödder, Veith, & Lötters, 2010; Olson et al., 2013). The Lower Guinean forests extend along western Central Africa and collectively host much of Africa's amphibian species richness and endemism (Jenkins, Pimm, & Joppa, 2013; Myers, Mittermeier, Mittermeier, da Fonseca, & Kent, 2000). This rich amphibian fauna includes hundreds of species with diverse life histories ranging from fully aquatic clawed frogs (*Xenopus*) to terrestrial leaf litter species that reproduce via direct development (*Arthroleptis*), and fossorial caecilians that give birth to live young (*Geotrypetes*). The Lower Guinean forests also extend to islands in the Gulf of Guinea archipelago (the land‐bridge island Bioko and the oceanic islands Príncipe, São Tomé, and Annobón), which differ in geologic histories, and consequently, in diversity and endemism of resident amphibians. Bioko Island is currently separated from adjacent Cameroon by ~30 km of shallow sea; however, historical cycles of rising and retreating sea levels resulted in periods of isolation and connectivity between Bioko and the adjacent continent (Meyers, Rosendahl, Harrison, & Ding, 1998). Consequently, Bioko Island hosts relatively high amphibian diversity for its size (~44 species of frogs and caecilians) and most of this diversity is also found in continental Guinean forests (Jones, 1994). By contrast, amphibians colonized the oceanic islands in the Gulf of Guinea via sweepstakes overseas dispersal (Bell et al., 2015; Measey et al., 2007), and thus, the islands host lower overall amphibian diversity: four frog and one caecilian species on São Tomé and three frog species on Príncipe, all of which are endemic (Bell, 2016; Jones, 1994; Uyeda, Drewes, & Zimkus, 2007). Recent surveys in Nigeria (Imasuen et al., 2011; Reeder, Cheng, Vredenburg, & Blackburn, 2011), Cameroon (Balaz, Kopecky, & Gvoždík, 2012; Doherty‐Bone et al., 2013; Hirschfeld et al., 2016), Gabon (Bell, Gata Garcia, Stuart, & Zamudio, 2011; Jongsma et al., 2016), and São Tomé Island (Hydeman, Bell, Drewes, & Zamudio, 2013) report *Bd* across a range of host species, elevations, and habitats in these assemblages. Thus, Lower Guinean forests present a unique opportunity to characterize *Bd* prevalence among related amphibian assemblages that naturally differ in species richness.

As in many infectious disease systems, a diverse assemblage of nonsusceptible amphibian hosts reduces *Bd* infection loads in experimental settings (Becker et al., 2014; Searle, Biga, Spatafora, & Blaustein, 2011; Venesky, Liu, Sauer, & Rohr, 2013). This outcome, termed the dilution effect (Keesing, Holt, & Ostfeld, 2006), is particularly likely when pathogen transmission is frequency‐dependent and noncompetent hosts are abundant and widespread. However, species richness is positively correlated with *Bd* occurrence in some wild populations (Becker & Zamudio, 2011), indicating that other factors such as environmental conditions and species identity likely alter disease outcomes. In particular, amphibian traits associated with pathogen exposure (e.g. aquatic index; Brem & Lips, 2008; Lips, Reeve, & Witters, 2003; Woodhams & Alford, 2005) and host competency (e.g. reservoirs and supershedders; DiRenzo, Langhammer, Zamudio, & Lips, 2014; Reeder, Pessier, & Vredenburg, 2012; Schloegel et al., 2010) may be predictably linked to community‐level disease dilution and amplification (Lloyd‐Smith, Schreiber, Kopp, & Getz, 2005; Streicker, Fenton, & Pedersen, 2013). Likewise, *Bd* prevalence varies with elevation (Brem & Lips, 2008), precipitation and temperature (Kielgast et al., 2010; Kriger & Hero, 2007; Longo et al., 2010), and forest canopy cover (Becker & Zamudio, 2011; Becker et al., 2012), all abiotic factors that are likely linked to the distribution of suitable cool and wet microhabitat conditions for the pathogen (Piotrowski, Annis, & Longcore, 2004).

Here, we survey *Bd* prevalence and genetic diversity across four biogeographically distinct amphibian assemblages. We use a combination of museum specimens and recent field surveys to (1) survey the prevalence of *Bd* in amphibian assemblages that differ in species composition and diversity and (2) characterize the strains of *Bd* present in each community. Specifically, we investigate whether *Bd* prevalence in amphibian communities is correlated with host diversity, host life history, and/or elevation. Based on intergenic transcribed spacer 1 (ITS1) haplotypes, we assess the diversity and identity of *Bd* in Central African Island and continental amphibian communities in comparison with known global *Bd* strains.

## 2 MATERIALS AND METHODS

### 2.1 Sampling and detection of *Bd*

We sampled 1027 amphibians (orders Gymnophiona and Anura) from three islands in the Gulf of Guinea Archipelago (Bioko, Príncipe and São Tomé) and continental Africa (Gabon) collected between 1935 and 2012 (Table S1). We sampled for *Bd* in the field in Gabon and São Tomé between 2009 and 2012 (308 amphibians) and in museum specimens collected on Bioko (*n* = 313), Príncipe (*n* = 130), and São Tomé (*n* = 276) between 1935 and 2012 (719 amphibians total). This sampling covers 11 amphibian families including all eight species that occur on São Tomé and Príncipe, ~50% of the 44 amphibian species reported from Bioko, and ~50% of the 96 amphibian species reported from Gabon (www.amphibiaweb.org). For amphibians sampled in the field, we captured frogs and caecilians by hand and placed them in individual plastic bags until processing. We collected samples from postmetamorphic individuals with sterile fine‐tip swabs (Medical Wire & Equipment Co. MW113) following the methods of Hyatt et al. (2007). Swabs were stored in 95% EtOH and kept as cool as possible in the field and then stored at −80°C until processing. The swabbed individuals (except *Amietophrynus superciliaris*, which is listed as CITES) were euthanized, prepared as voucher specimens (Table S1), and deposited at the Cornell University Museum of Vertebrates (CUMV), the Museum of Comparative Zoology at Harvard University (MCZ), the California Academy of Sciences (CAS), and North Carolina Museum of Natural Sciences (NCSM). For amphibians sampled as museum specimens, the vast majority (716/719) were collected between 1998 and 2012, formalin‐preserved at the time of collection, and stored in 70% ethanol (range: 1–101 specimens per jar). The original collection and storage methods for the pre‐1998 specimens are unknown, but at time of sampling they were stored in (70%) ethanol. Museum specimens were rinsed with clean 70% ethanol and swabbed with sterile fine‐tip swabs (Medical Wire & Equipment Co. MW113) following standard procedures for preserved specimens (Cheng, Rovito, Wake, & Vredenburg, 2011; Hyatt et al., 2007). All swabs were stored in 95% EtOH at 4°C until processing.

We followed established methods for DNA extraction and quantitative *Bd* detection in the laboratory (Boyle, Boyle, Olsen, Morgan, & Hyatt, 2004). Briefly, we extracted DNA from each swab using 50 μl of Prepman Ultra and detected the presence of *Bd* with duplicate qPCRs (Boyle et al., 2004), performed using Taqman Fast Advanced Master Mix on a ViiA7 Real‐Time PCR System (Applied Biosystems, Carlsbad, CA, USA). Samples that showed signs of inhibition (nonsigmoidal amplification) were further diluted to 1:100 and re‐analyzed. All samples were evaluated in duplicate plates. We generated standard curves from templates of known zoospore concentrations of *Bd* strain JEL427 (Puerto Rico, Luquillo) ranging from 0.1 to 1,000 zoospores (Boyle et al., 2004). To evaluate fluorescence levels of the samples and standards, we used ViiA 7 software (Applied Biosystems). For swabs collected from amphibians in the field, we deemed samples positive when significant sigmoidal amplification genomic equivalents (GE) (GE ≥ 1) occurred in one or both qPCR reactions. For museum specimens, we deemed samples with *C*_t_ <40 (equivalent to GE ≥ 1) in both qPCR replicates as positive (Kriger & Hero, 2007; Rodriguez et al., 2014). We do not report infection intensity because ITS1 copy number variation among *Bd* strains directly influences qPCR estimates of pathogen load (Longo et al., 2013) and the copy number variation of Central African *Bd* strains is unknown. Furthermore, the effects of preservation on estimating pathogen loads from museum specimens are poorly understood. Results of the field‐sampled *Bd* surveys from 2009 (Gabon) and 2012 (São Tomé) were previously reported in Bell et al. (2011) and Hydeman et al. (2013), respectively.

### 2.2 Infection prevalence analyses

For species and localities with sample sizes >20, we estimated *Bd* prevalence by dividing the number of positive individuals by the total number of individuals sampled and estimated 95% Clopper‐Pearson confidence intervals (α = 0.05). To determine whether breeding biology and life history are correlated with *Bd* prevalence, we calculated the lifetime aquatic index for each species in our dataset (Lips et al., 2003; Table S1) and tested for a significant relationship between *Bd* prevalence and aquatic index using linear regression. To test for the dilution effect across amphibian communities that differ in species richness, we estimated *Bd* prevalence in localities with sample sizes >20 (field and museum surveys) and tested for a significant relationship between *Bd* prevalence and amphibian community species richness, average aquatic index, and elevation using multiple regression. Seven of the 16 sites included samples grouped across multiple survey years. Jongsma et al. (2016) surveyed *Bd* prevalence across seven amphibian communities in Gabon using similar field and laboratory detection methods; therefore, for comparison we included localities from the Jongsma et al. surveys with sample sizes greater than 20 (six communities) in the regression analysis. We conducted all statistical analyses in R version 3.1.3 R Core Team (2015).

### 2.3 ITS1 haplotype sequencing and diversity

Following Goka et al. (2009) and Rodriguez et al. (2014), we used a semi‐nested PCR approach to generate amplicons for cloning and sequencing approximately 150 bp of the ITS1 region for 44 samples that exhibited amplification curves in the qPCR analyses. For one subset of samples, we performed the first PCR using primers ITS1‐3 Chytr (Boyle et al., 2004) and Bd2a (Annis, Dastoor, Ziel, Daszak, & Longcore, 2004), used 1 μl of the PCR product as template for a second PCR using primers 5.8S Chytr and ITS1‐3 Chytr (Boyle et al., 2004), and both PCRs used a touchdown thermal profile with negative controls. For the second subset of samples, we performed the first PCR using primers Bd18SF1 and Bd28SR1 (Goka et al., 2009), used 1 μl of the PCR product as template for a second PCR using primers Bd1a and Bd2a (Annis et al., 2004), and cycling conditions followed (Goka et al., 2009) with both negative and positive controls. PCR products were visualized on an agarose gel, purified using ExoSAP‐IT (USB Corp., Cleveland, OH, USA), and sequenced using a BigDye Terminator Cycle Sequencing Kit v.3.1 (Applied Biosystems, Foster City, CA, USA) on an ABI Automated 3730xl Genetic Analyzer (Applied Biosystems). For eight of the successful PCRs, we cloned the products into JM109‐competent cells following the manufacturer's instructions for the pGEM‐T Easy Vector System I (Promega Inc.) and used blue/white screening to identify transformed colonies. We placed colonies in 25 μl of ddH_2_O, incubated them at 95°C for 10 min, performed a final amplification using M13 primers, and verified successful transformations by electrophoresis on a 1.75% agarose gel. We purified amplicons using ExoSAP‐IT and sequenced them using a BigDye Terminator Cycle Sequencing Kit v.3.1 with the M13 primer on an ABI Automated 3730xl Genetic Analyzer. We edited chromatograms from the combined 44 *Bd*‐positive samples using SEQUENCHER 5.1 (GeneCodes, Inc.).

Strains of *Bd* differ in number and identity of ITS1 haplotypes (Longo et al., 2013); therefore, interpreting evolutionary relationships among strains using a typical phylogenetic approach can be misleading. Instead, we compared ITS1 haplotypes recovered in our study to published reference strains (Longo et al., 2013; Rodriguez et al., 2014) and genome sequences (Farrer et al., 2011; Rosenblum et al., 2013) to determine whether the strains of *Bd* present at our sample sites likely belong to *Bd*GPL, other previously identified strains of *Bd* (e.g. *Bd*Brazil, *Bd*Cape), or novel strains. To visualize the overall diversity, abundance, and geographic distribution of haplotypes we recovered in this study, we created a haplotype network using TCS v 1.21 (Clement, Posada, & Crandall, 2000).

## 3 RESULTS

### 3.1 Bd prevalence

**Figure 1:**
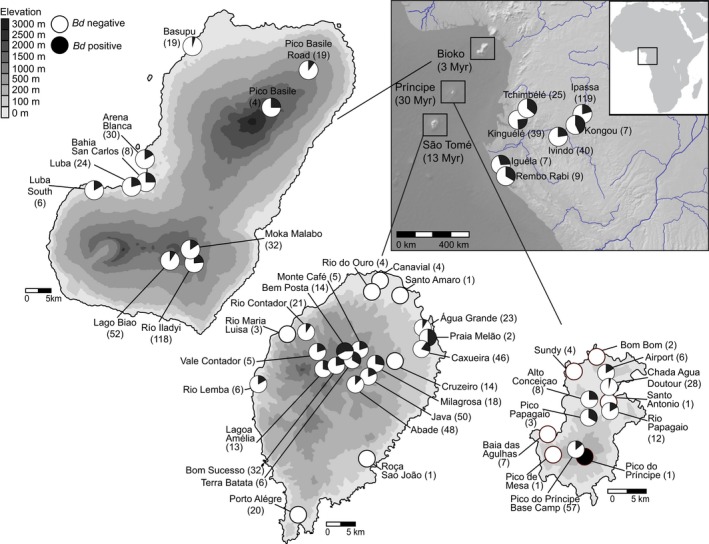
Sampling localities and *Bd* prevalence in Gabon, Bioko Island, and São Tomé and Príncipe, Africa. Sample size of amphibians swabbed per site is indicated in parentheses

We detected *Bd* at 39 of the 50 sites in Gabon and the Gulf of Guinea Archipelago (Figure 1) with an overall prevalence of 18.9% (16.4%–21.3% confidence limit) and a range of 0% to 25.6% prevalence for sites with sample sizes >20 individuals (Table 1). For the museum specimens, an *Amietophrynus camerunensis* collected on Bioko Island in 1966 was our earliest positive sample while both of the pre‐2001 samples from São Tomé were negative (*Schistometopum thomense* collected in 1935 and 1949). The earliest positive samples for São Tomé and Príncipe are from the first California Academy of Sciences expedition to the islands in 2001. We detected *Bd* across all subsequent sample years in our study (Table S1).

**Table 1:** Overall *Bd* prevalence and elevational range for island and continental amphibian communities ”in bold”, and local *Bd* prevalence for sampling localities with sample sizes >20 individuals

Sampling Locality	NS	NI/N	Prevalence (%)	95% CI	Elevation (m)
**Bioko**	**21**	**56/313**	**17.9**	**13.8–22.6**	**2**–**1,870**
Bioko—Arena Blanca	2	5/30	16.7	5.6–34.7	29–76
Bioko—Lago Biao	3	5/52	9.6	3.2–21.0	1,860–1,870
Bioko—Moka Malabo	8	5/32	15.6	5.3–32.8	1264–1,414
Bioko—Moka, Río Iladyi	8	29/119	24.4	17.0–33.1	1,143–1,291
**São Tomé**	**5**	**60/338**	**17.8**	**13.8–22.3**	**11**–**1,444**
São Tomé—Abade	2	6/48	12.5	4.7–25.2	400–688
São Tomé—Água Grande	2	2/23	8.7	1.1–28.0	11
São Tomé—Bom Sucesso	4	7/32	21.9	9.3–40.0	1,156–1,326
São Tomé—Caxueira	4	9/46	19.6	9.4–33.9	49–65
São Tomé—Java	3	11/50	22.0	11.5–36.0	592–600
São Tomé—Porto Alegre	1	0/20	0	0–16.8	18
São Tomé—Rio Contador	2	2/21	9.5	1.2–30.4	619
**Gabon** a	**46**	**61/246**	**24.8**	**19.5–30.7**	**7**–**565**
Gabon— Ivindo National Park, Ipassa Station	28	25/119	21.0	14.1–29.4	480–545
Gabon—Ivindo, Rougier Forestry Concession	10	9/40	22.5	10.8–38.5	188–276
Gabon—Monts de Cristal, Kinguélé	23	10/39	25.6	13.0–42.1	65–186
Gabon—Mitone^b^	26	29/110	26.4	18.4–35.6	43
Gabon—Carivenville^b^	18	19/71	26.8	16.9–38.6	44
Gabon—Junkville^b^	17	10/99	10.1	4.9–17.8	86
Gabon—Madoukou^b^	10	11/35	31.4	16.9–49.3	246
Gabon—Mboua^b^	6	5/33	15.2	5.1–31.9	504
Gabon—Doumaye^b^	22	9/105	8.6	4.0–15.7	526
**Príncipe**	**3**	**16/130**	**12.3**	**7.2–19.2**	**17**–**950**
Príncipe—Agua Doutor	2	1/28	3.6	0.1–18.4	178
Príncipe— Pico do Príncipe, Base Camp	2	8/57	14.0	6.3–25.8	357–620

NS, number of species sampled; NI/N, infected individuals/total individuals sampled; Clopper‐Pearson confidence intervals for species level prevalence (α = 0.05).

a Gabon totals include sampling from Bell et al., 2011 and this study.

b Data from Jongsma et al., 2016.

**Figure 2:**
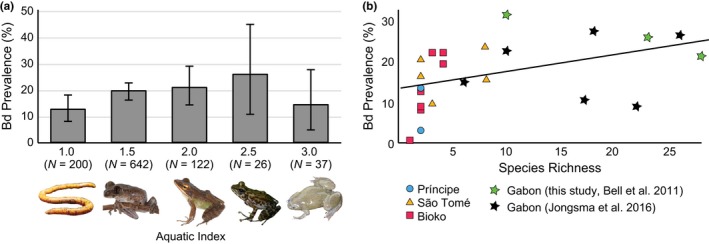
(a) *Bd* prevalence (Clopper‐Pearson confidence intervals α = 0.05) with respect to lifetime aquatic index. Representative species for each class of lifetime aquatic index: *Schistometopum thomensis*,* Leptopelis aubryi*,* Hylarana albolabris*,* Petropedetes palmipes*, and *Silurana epitropicalis* (Photos A. Stanbridge and B. Stuart). (b) *Bd* prevalence with respect to species richness in 23 amphibian communities sampled in Príncipe (blue circles), São Tomé (yellow triangles), Bioko (red squares), and Gabon (stars)

The amphibian communities we sampled include representative species with a lifetime aquatic index of 1 (e.g., the live‐bearing terrestrial caecilian *Schistometopum thomense)* through 3 (e.g., the fully aquatic African clawed frogs, *Silurana epitropicalis*; Table S1). We recovered a trend of increasing prevalence with higher lifetime aquatic index from 1 to 2.5 (Figure 2a); however, the trend does not continue with aquatic index 3 (African clawed frogs) and is therefore not significant (*p* > .05). Species richness was a significant predictor of amphibian community estimates of *Bd* prevalence (*p* < .05, Figure 2b); *Bd* prevalence increased with species richness. We did not find a relationship between *Bd* prevalence in amphibian communities and elevation (*p* > .05).

### 3.2 ITS1 haplotype diversity

**Figure 3:**
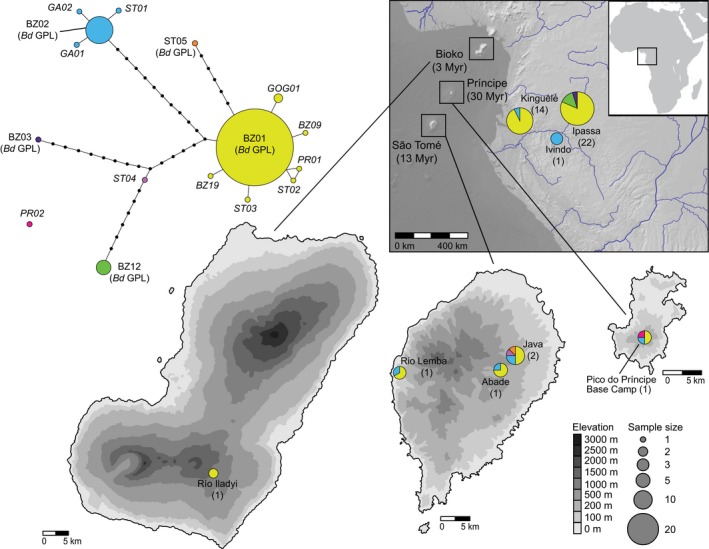
Haplotype network and distribution of *ITS* haplotypes sequenced from *Bd*‐positive amphibians in Gabon, Equatorial Guinea (Bioko Island), and São Tomé and Príncipe, Africa. Sample size of *Bd*‐positive amphibians (anurans and caecilians) sequenced for *ITS* per locality indicated in parentheses. The PR02 haplotype is disconnected from the rest of the network because it requires more than 10 steps to connect this haplotype to the remaining haplotypes

We successfully sequenced ITS1 from 36 *Bd*‐positive samples using the nested PCR approaches and obtained multiple ITS1 clones from an additional eight *Bd*‐positive samples (6 clones per sample) resulting in 1–5 unique ITS1 haplotypes per sample. We found 16 unique ITS1 haplotypes across the 44 sequenced *Bd*‐positive samples, the most common of which were BZhap01 and BZhap02 (names follow Rodriguez et al., 2014). These two haplotypes are members of the *Bd*GPL lineage and were present at the eight sites for which we obtained *Bd* sequences, which include localities in Bioko, São Tomé, Príncipe, and Gabon (Figure 3, Table S2). Four of the remaining low‐frequency haplotypes we recovered are identical to those reported in previous surveys of ITS1 haplotype diversity in the neotropics (Longo et al., 2013; Rodriguez et al., 2014). We also recovered nine novel haplotypes with high sequence similarity to *Bd*GPL haplotypes sequenced in previous studies (0.6%–4.5% sequence divergence) and one highly divergent haplotype (PR02; 15.4% sequence divergence) that does not match any of the *Bd* genomes sequenced to date. The ITS alignment is available in the online supporting information.

## 4 DISCUSSION

The overall prevalence of *Bd* in the amphibian communities we sampled is similar to that of surveys in recent and historical *Bd*‐positive communities in continental Africa and is consistent with a long‐term and widespread distribution of *Bd* in African amphibians (Doherty‐Bone et al., 2013; Jongsma et al., 2016; Kielgast et al., 2010; Soto‐Azat et al., 2010; Weldon, Du Preez, Hyatt, Muller, & Speare, 2004). *Bd* has yet to be detected in wild amphibian populations west of the Dahomey Gap in West Africa (Penner et al., 2013) or in the Seychelles (Labisko et al., 2015) despite a diversity of potential hosts and suitable environmental conditions. This pattern may reflect prominent biogeographic barriers (the Indian Ocean and the dry forest‐savannah mosaic across the Dahomey Gap) that delimit distinct amphibian assemblages, and thus potentially restrict *Bd* dispersal. Our results confirm that *Bd* successfully colonized the land‐bridge island Bioko and the oceanic islands of São Tomé and Príncipe, although it is unclear whether the pathogen arrived with its hosts or independently colonized the islands. Furthermore, although our sampling of museum specimens included only a few individuals collected in the Gulf of Guinea archipelago prior to 1998 because few historical samples are available in museum collections, one of these individuals was *Bd*‐positive indicating that the pathogen has been on Bioko for at least 50 years. Surveys for *Bd* in Madagascar over the last 10 years first detected the pathogen in 2010; however, it is unclear whether *Bd* only recently colonized the island or if seasonal variation in detection probability produced false negative results in earlier surveys (Bletz et al., 2015). Thus, limited sampling and seasonal variation may also explain negative results of *Bd* surveys in West Africa and in some regions of Gabon (Daversa, Bosch, & Jeffery, 2011; Gratwicke et al., 2011; Penner et al., 2013).

Although infection intensity is a key component of *Bd*‐amphibian disease dynamics (Becker et al., 2014; Vredenburg et al., 2010), the effects of specimen preservation and unknown ITS copy number in Central African *Bd* preclude us from estimating pathogen load in our samples. Thus, we focused on whether *Bd* prevalence in amphibian communities is correlated with species life history, species richness, and/or elevation. The emerging consensus in the *Bd* literature is that life history traits such as aquatic index are important predictors of susceptibility (James et al., 2015), yet we found modest support for a relationship between aquatic index and *Bd* prevalence in Central African amphibians. *Bd* prevalence in terrestrial species that reproduce via direct development or give birth to live young (aquatic index = 1) was lower than that of riparian species with aquatic larvae (aquatic index = 2.5) but comparable to species that are fully aquatic (aquatic index = 3). This pattern may be due in part to taxonomic bias in our sampling of fully aquatic species, which was primarily represented by the genera *Silurana* and *Xenopus* that typically exhibit low prevalence and infection intensity in natural populations (Kielgast et al., 2010; Soto‐Azat et al., 2010; Weldon et al., 2004). Riparian species with aquatic larvae (aquatic index = 2.5) have high infection intensities in field surveys in Kenya (Kielgast et al., 2010) and Gabon (Jongsma et al., 2016) indicating that species with these life histories are highly susceptible to *Bd* infections. Although no studies to date have linked African amphibian declines to chytridiomycosis and individuals with high *Bd* loads appear asymptomatic (Jongsma et al., 2016; Kielgast et al., 2010), studies in Central American, South American, and Australian amphibian communities demonstrate that species with aquatic larvae are more likely to decline (Carvalho, Becker, & Toledo, 2017; Hero & Morrison, 2004; Lips et al., 2008), and those that are tolerant may spread *Bd* between aquatic and terrestrial habitats (Brem & Lips, 2008). Our results indicate that there is potential for these same disease dynamics to operate in Central African amphibian communities.

The dilution effect, whereby a diverse assemblage of amphibian hosts reduces *Bd* infection, is particularly likely when pathogen transmission is frequency‐dependent and the most abundant and widespread species are noncompetent hosts (Keesing et al., 2006). Our surveys of *Bd* prevalence reveal that amphibian communities in the Lower Guinean forests include many competent and widely distributed host species, and correspondingly, we found a positive relationship between *Bd* prevalence and species richness. This pattern of pathogen augmentation is consistent with field studies of *Bd* prevalence in Costa Rica and Australia (Becker & Zamudio, 2011), indicating that dilution and amplification of pathogen prevalence in wild populations may be predictable based on host species traits (Venesky et al., 2013). We did not find a significant relationship between elevation and *Bd* prevalence across our sample sites; however, our sampling was biased to primarily lowland sites and does not include as wide an elevational range as previous studies in Central and East Africa that found a significantly higher *Bd* prevalence at higher elevations (Hirschfeld et al., 2016; Kielgast et al., 2010).

DNA sequencing of the pathogen revealed that *Bd*GPL—the most widespread and virulent lineage (Farrer et al., 2011)—is present in all four amphibian assemblages we sampled, and we did not recover any haplotypes indicative of other global *Bd* lineages sequenced to date. Although the presence of *Bd*GPL is often equated with a recent invasion of this virulent lineage, we recovered high haplotype diversity across our modest sample size, including a number of low‐frequency haplotypes identified in previous studies (Longo et al., 2013; Rodriguez et al., 2014) as well as several novel haplotypes. This result, along with our early record of *Bd* on Bioko Island (1966) indicates that *Bd*GPL likely has a historical presence in continental Central Africa and the Gulf of Guinea islands. Characterizing *Bd* lineages present in other African amphibian assemblages is an important next step for understanding the history of this pathogen across the continent and will be essential for predicting whether *Bd* poses a threat to African amphibians.

## AUTHOR CONTRIBUTIONS

M.E.H. and R.C.B designed the project; R.C.B. collected data from field samples; M.E.H. collected data from museum samples; M.E.H., A.V.L., G.V.A., D.R., and R.C.B. collected and analyzed the data; M.E.H., R.C.B., and K.R.Z. contributed funding to the project; M.E.H. and R.C.B. wrote the manuscript with input from all authors.

## CONFLICT OF INTEREST

None declared.
