# Supplementary material for: Prevalence and genetic diversity of Batrachochytrium dendrobatidis in Central African island and continental amphibian communities
Source: Ecol Evol. 2017 Aug 22;7(19):7729–38. doi: 10.1002/ece3.3309 (PMC5632636; doi:10.1002/ece3.3309)
Supplement: Supplementary file 1 [file ECE3-7-7729-s001.pdf]

Table S1: Sampling localities, *Bd* screening result, and voucher information for retrospective and contemporary *Bd* surveys. All samples from Bell *et al.* 2011 and Hydeman e*t al.* 2013 were field-caught specimens. Abbreviations as follow: Equatorial Guinea - Bioko Island (EGBK), Gabon - Estuaire (GAES), Gabon - Ogooué-Maritime (GAOM), Gabon - Ogooué-Ivindo (GAOI), Gabon - Woleu-Ntem (WAWN), São Tomé + Príncipe - São Tomé Island (STST), São Tomé + Príncipe - Príncipe Island (STPR), Cornell University Museum of Vertebrates (CU), the California Academy of Sciences (CAS), the North Carolina Museum of Natural Sciences (NCSM), and the Museum of Comparative Zoology (MCZ).

Species	Cat. No.	Aq. Index	Country	Locality	Elevation (m)	Lat	Long	Collected	*Bd*	Collection Method
Afrixalus laevis	CAS 207542	1.5	EGBK	Moka, Río Iladyi	1143	3.325	8.671	1-Oct-98		Museum Specimen
Afrixalus laevis	CAS 207543	1.5	EGBK	Moka, Río Iladyi	1143	3.325	8.671	1-Oct-98	(+)	Museum Specimen
Afrixalus laevis	CAS 207544	1.5	EGBK	Moka, Río Iladyi	1143	3.325	8.671	1-Oct-98		Museum Specimen
Afrixalus laevis	CAS 207545	1.5	EGBK	Moka, Río Iladyi	1143	3.325	8.671	1-Oct-98		Museum Specimen
Afrixalus laevis	CAS 207546	1.5	EGBK	Moka, Río Iladyi	1143	3.325	8.671	1-Oct-98	(+)	Museum Specimen
Afrixalus laevis	CAS 207547	1.5	EGBK	Moka, Río Iladyi	1143	3.325	8.671	1-Oct-98	(+)	Museum Specimen
Afrixalus laevis	CAS 207548	1.5	EGBK	Moka, Río Iladyi	1143	3.325	8.671	1-Oct-98	(+)	Museum Specimen
Afrixalus laevis	CAS 207549	1.5	EGBK	Moka, Río Iladyi	1143	3.325	8.671	1-Oct-98		Museum Specimen
Afrixalus laevis	CAS 207550	1.5	EGBK	Moka, Río Iladyi	1143	3.325	8.671	1-Oct-98	(+)	Museum Specimen
Afrixalus laevis	CAS 207551	1.5	EGBK	Moka, Río Iladyi	1143	3.325	8.671	1-Oct-98	(+)	Museum Specimen
Afrixalus laevis	CAS 207552	1.5	EGBK	Moka, Río Iladyi	1143	3.325	8.671	1-Oct-98		Museum Specimen
Afrixalus laevis	CAS 207553	1.5	EGBK	Moka, Río Iladyi	1143	3.325	8.671	1-Oct-98		Museum Specimen
Afrixalus laevis	CAS 207554	1.5	EGBK	Moka, Río Iladyi	1143	3.325	8.671	1-Oct-98		Museum Specimen
Afrixalus laevis	CAS 207555	1.5	EGBK	Moka, Río Iladyi	1143	3.325	8.671	1-Oct-98		Museum Specimen
Afrixalus laevis	CAS 207556	1.5	EGBK	Moka, Río Iladyi	1143	3.325	8.671	1-Oct-98		Museum Specimen
Afrixalus laevis	CAS 207557	1.5	EGBK	Moka, Río Iladyi	1143	3.325	8.671	1-Oct-98		Museum Specimen
Afrixalus laevis	CAS 207558	1.5	EGBK	Moka, Río Iladyi	1143	3.325	8.671	1-Oct-98		Museum Specimen
Afrixalus laevis	CAS 207559	1.5	EGBK	Moka, Río Iladyi	1143	3.325	8.671	1-Oct-98		Museum Specimen
Afrixalus laevis	CAS 207560	1.5	EGBK	Moka, Río Iladyi	1143	3.325	8.671	1-Oct-98		Museum Specimen
Afrixalus laevis	CAS 207561	1.5	EGBK	Moka, Río Iladyi	1143	3.325	8.671	1-Oct-98		Museum Specimen
Afrixalus laevis	CAS 207562	1.5	EGBK	Moka, Río Iladyi	1143	3.325	8.671	1-Oct-98		Museum Specimen
Afrixalus laevis	CAS 207563	1.5	EGBK	Moka, Río Iladyi	1143	3.325	8.671	1-Oct-98		Museum Specimen
Afrixalus laevis	CAS 207564	1.5	EGBK	Moka, Río Iladyi	1143	3.325	8.671	1-Oct-98		Museum Specimen
Afrixalus laevis	CAS 207565	1.5	EGBK	Moka, Río Iladyi	1143	3.325	8.671	1-Oct-98		Museum Specimen
Afrixalus laevis	CAS 207566	1.5	EGBK	Moka, Río Iladyi	1143	3.325	8.671	5-Oct-98		Museum Specimen
Afrixalus laevis	CAS 207567	1.5	EGBK	Moka, Río Iladyi	1143	3.325	8.671	5-Oct-98		Museum Specimen
Afrixalus laevis	CAS 207568	1.5	EGBK	Moka, Río Iladyi	1143	3.325	8.671	6-Oct-98		Museum Specimen
Afrixalus laevis	CAS 207569	1.5	EGBK	Moka, Río Iladyi	1143	3.325	8.671	6-Oct-98		Museum Specimen
Afrixalus paradorsalis	CAS 207523	1.5	EGBK	Moka, Río Iladyi	1143	3.325	8.671	1-Oct-98		Museum Specimen
Afrixalus paradorsalis	CAS 207524	1.5	EGBK	Moka, Río Iladyi	1143	3.325	8.671	1-Oct-98		Museum Specimen
Afrixalus paradorsalis	CAS 207525	1.5	EGBK	Moka, Río Iladyi	1143	3.325	8.671	1-Oct-98		Museum Specimen
Afrixalus paradorsalis	CAS 207526	1.5	EGBK	Moka, Río Iladyi	1143	3.325	8.671	1-Oct-98		Museum Specimen
Afrixalus paradorsalis	CAS 207527	1.5	EGBK	Moka, Río Iladyi	1143	3.325	8.671	1-Oct-98		Museum Specimen
Afrixalus paradorsalis	CAS 207528	1.5	EGBK	Moka, Río Iladyi	1143	3.325	8.671	1-Oct-98		Museum Specimen
Afrixalus paradorsalis	CAS 207529	1.5	EGBK	Moka, Río Iladyi	1143	3.325	8.671	1-Oct-98		Museum Specimen
Afrixalus paradorsalis	CAS 207530	1.5	EGBK	Moka, Río Iladyi	1143	3.325	8.671	1-Oct-98		Museum Specimen
Afrixalus paradorsalis	CAS 207531	1.5	EGBK	Moka, Río Iladyi	1143	3.325	8.671	1-Oct-98		Museum Specimen
Afrixalus paradorsalis	CAS 207532	1.5	EGBK	Moka, Río Iladyi	1143	3.325	8.671	1-Oct-98		Museum Specimen
Afrixalus paradorsalis	CAS 207533	1.5	EGBK	Moka, Río Iladyi	1143	3.325	8.671	1-Oct-98		Museum Specimen
Afrixalus paradorsalis	CAS 207534	1.5	EGBK	Moka, Río Iladyi	1143	3.325	8.671	1-Oct-98		Museum Specimen
Afrixalus paradorsalis	CAS 207535	1.5	EGBK	Moka, Río Iladyi	1143	3.325	8.671	1-Oct-98		Museum Specimen
Afrixalus paradorsalis	CAS 207536	1.5	EGBK	Moka, Río Iladyi	1143	3.325	8.671	1-Oct-98		Museum Specimen
Afrixalus paradorsalis	CAS 207537	1.5	EGBK	Moka, Río Iladyi	1143	3.325	8.671	1-Oct-98		Museum Specimen
Afrixalus paradorsalis	CAS 207538	1.5	EGBK	Moka, Río Iladyi	1143	3.325	8.671	1-Oct-98		Museum Specimen
Afrixalus paradorsalis	CAS 207539	1.5	EGBK	Moka, Río Iladyi	1143	3.325	8.671	6-Oct-98		Museum Specimen
Afrixalus paradorsalis	CAS 207540	1.5	EGBK	Moka, Río Iladyi	1143	3.325	8.671	6-Oct-98		Museum Specimen
Afrixalus paradorsalis	CAS 207541	1.5	EGBK	Moka, Río Iladyi	1143	3.325	8.671	6-Oct-98		Museum Specimen
Afrixalus paradorsalis	CAS 207570	1.5	EGBK	Luba	2	3.461	8.552	12-Oct-98		Museum Specimen
Afrixalus paradorsalis	CAS 207571	1.5	EGBK	Luba	2	3.461	8.552	12-Oct-98		Museum Specimen
Afrixalus paradorsalis	CAS 207572	1.5	EGBK	Luba	2	3.461	8.552	12-Oct-98		Museum Specimen
Afrixalus paradorsalis	CAS 207573	1.5	EGBK	Luba	2	3.461	8.552	12-Oct-98		Museum Specimen
Afrixalus paradorsalis	CAS 207574	1.5	EGBK	Luba	2	3.461	8.552	12-Oct-98		Museum Specimen
Afrixalus paradorsalis	CAS 207575	1.5	EGBK	Luba	2	3.461	8.552	12-Oct-98		Museum Specimen
Afrixalus paradorsalis	CAS 207576	1.5	EGBK	Luba	2	3.461	8.552	12-Oct-98		Museum Specimen
Afrixalus paradorsalis	CAS 207577	1.5	EGBK	Luba	2	3.461	8.552	12-Oct-98		Museum Specimen
Afrixalus paradorsalis	CAS 207578	1.5	EGBK	Luba	2	3.461	8.552	12-Oct-98		Museum Specimen
Afrixalus paradorsalis	CAS 207579	1.5	EGBK	Luba	2	3.461	8.552	12-Oct-98		Museum Specimen
Afrixalus paradorsalis	CAS 207580	1.5	EGBK	Luba	2	3.461	8.552	12-Oct-98		Museum Specimen
Afrixalus paradorsalis	CAS 207581	1.5	EGBK	Luba	2	3.461	8.552	12-Oct-98		Museum Specimen
Afrixalus paradorsalis	CAS 207582	1.5	EGBK	Luba	2	3.461	8.552	12-Oct-98		Museum Specimen
Afrixalus paradorsalis	CAS 207583	1.5	EGBK	Luba	2	3.461	8.552	12-Oct-98		Museum Specimen
Afrixalus paradorsalis	CAS 207584	1.5	EGBK	Luba	2	3.461	8.552	12-Oct-98	(+)	Museum Specimen
Afrixalus paradorsalis	CAS 207585	1.5	EGBK	Luba	2	3.461	8.552	12-Oct-98		Museum Specimen
Afrixalus paradorsalis	CAS 207586	1.5	EGBK	Luba	2	3.461	8.552	12-Oct-98		Museum Specimen
Afrixalus paradorsalis	CAS 207587	1.5	EGBK	Luba	2	3.461	8.552	12-Oct-98		Museum Specimen
Afrixalus paradorsalis	CAS 207588	1.5	EGBK	Pico Basile rd	548	3.705	8.879	18-Oct-98		Museum Specimen
Afrixalus paradorsalis	CAS 207589	1.5	EGBK	Pico Basile rd	548	3.705	8.879	18-Oct-98		Museum Specimen
Afrixalus paradorsalis	CAS 207590	1.5	EGBK	Basupu	57	3.711	8.667	18-Oct-98		Museum Specimen
Afrixalus paradorsalis	CAS 207591	1.5	EGBK	Basupu	57	3.711	8.667	18-Oct-98		Museum Specimen
Afrixalus paradorsalis	CAS 207592	1.5	EGBK	Basupu	57	3.711	8.667	18-Oct-98		Museum Specimen
Afrixalus paradorsalis	CAS 207593	1.5	EGBK	Basupu	57	3.711	8.667	18-Oct-98		Museum Specimen
Afrixalus paradorsalis	CAS 207594	1.5	EGBK	Basupu	57	3.711	8.667	18-Oct-98		Museum Specimen
Afrixalus paradorsalis	CAS 207595	1.5	EGBK	Basupu	57	3.711	8.667	18-Oct-98		Museum Specimen
Afrixalus paradorsalis	CAS 207596	1.5	EGBK	Basupu	57	3.711	8.667	18-Oct-98		Museum Specimen
Afrixalus paradorsalis	CAS 207597	1.5	EGBK	Basupu	57	3.711	8.667	18-Oct-98		Museum Specimen
Afrixalus paradorsalis	CAS 207598	1.5	EGBK	Basupu	57	3.711	8.667	18-Oct-98		Museum Specimen
Amietophrynus camerunensis	CAS 103730	1.5	EGBK	Road to Moka	unknown	N/A	N/A	8-Mar-66	(+)	Museum Specimen

Species	Cat. No.	Aq. Index	Country	Locality	Elevation (m)	Lat	Long	Collected	*Bd*	Collection Method
Amietophrynus camerunensis	CAS 207288	1.5	EGBK	Moka Malabo	1414	3.361	8.662	30-Sep-98	(+)	Museum Specimen
Amietophrynus camerunensis	CAS 207289	1.5	EGBK	Moka Malabo	1414	3.361	8.662	30-Sep-98	(+)	Museum Specimen
Amietophrynus camerunensis	CAS 207290	1.5	EGBK	Moka Malabo	1414	3.361	8.662	1-Oct-98	(+)	Museum Specimen
Amietophrynus camerunensis	CAS 207291	1.5	EGBK	Moka Malabo	1414	3.361	8.662	1-Oct-98	(+)	Museum Specimen
Amietophrynus camerunensis	CAS 207292	1.5	EGBK	Moka Malabo	1414	3.361	8.662	2-Oct-98		Museum Specimen
Amietophrynus camerunensis	CAS 207293	1.5	EGBK	Moka Malabo	1414	3.361	8.662	2-Oct-98		Museum Specimen
Amietophrynus camerunensis	CAS 207294	1.5	EGBK	Moka Malabo	1414	3.361	8.662	2-Oct-98		Museum Specimen
Amietophrynus camerunensis	CAS 207295	1.5	EGBK	Moka Malabo	1414	3.361	8.662	2-Oct-98		Museum Specimen
Amietophrynus camerunensis	CAS 207690	1.5	EGBK	Lago Biao	1860	3.355	8.622	9-Oct-98		Museum Specimen
Amietophrynus camerunensis	CAS 207691	1.5	EGBK	Lago Biao	1860	3.355	8.622	9-Oct-98		Museum Specimen
Amietophrynus camerunensis	CAS 207692	1.5	EGBK	Lago Biao	1860	3.355	8.622	9-Oct-98		Museum Specimen
Amietophrynus camerunensis	CAS 207693	1.5	EGBK	Lago Biao	1860	3.355	8.622	9-Oct-98		Museum Specimen
Amietophrynus camerunensis	CAS 207694	1.5	EGBK	Lago Biao	1860	3.355	8.622	9-Oct-98		Museum Specimen
Amietophrynus camerunensis	CAS 207695	1.5	EGBK	Lago Biao	1860	3.355	8.622	9-Oct-98		Museum Specimen
Amietophrynus camerunensis	CAS 207696	1.5	EGBK	Lago Biao	1860	3.355	8.622	9-Oct-98		Museum Specimen
Amietophrynus camerunensis	CAS 207697	1.5	EGBK	Lago Biao	1860	3.355	8.622	9-Oct-98		Museum Specimen
Amietophrynus camerunensis	CAS 207698	1.5	EGBK	Lago Biao	1860	3.355	8.622	9-Oct-98		Museum Specimen
Amietophrynus camerunensis	CAS 207699	1.5	EGBK	Lago Biao	1860	3.355	8.622	9-Oct-98		Museum Specimen
Amietophrynus camerunensis	CAS 207700	1.5	EGBK	Lago Biao	1860	3.355	8.622	9-Oct-98		Museum Specimen
Amietophrynus camerunensis	CAS 207701	1.5	EGBK	Lago Biao	1860	3.355	8.622	9-Oct-98		Museum Specimen
Amietophrynus camerunensis	CAS 207702	1.5	EGBK	Lago Biao	1860	3.355	8.622	9-Oct-98		Museum Specimen
Amietophrynus camerunensis	CAS 207944	1.5	EGBK	Lago Biao	1870	3.352	8.635	9-Oct-98		Museum Specimen
Amietophrynus camerunensis	CAS 207951	1.5	EGBK	Moka Malabo	1414	3.361	8.667	3-Oct-98		Museum Specimen
Amietophrynus camerunensis	CAS 207952	1.5	EGBK	Moka Malabo	1414	3.361	8.667	3-Oct-98		Museum Specimen
Amietophrynus camerunensis	CAS 207953	1.5	EGBK	Moka, Río Iladyi	1143	3.325	8.671	5-Oct-98		Museum Specimen
Amietophrynus camerunensis	CAS 207954	1.5	EGBK	Moka, Río Iladyi	1291	3.336	8.666	6-Oct-98		Museum Specimen
Amietophrynus camerunensis	CAS 207955	1.5	EGBK	Moka, Río Iladyi	1291	3.336	8.666	6-Oct-98		Museum Specimen
Amietophrynus gracilipes	CAS 207620	2	EGBK	Basupu	57	3.711	8.667	18-Oct-98		Museum Specimen
Arthroleptis bioko	CAS 207282	1	EGBK	Pico Basile	1819	3.628	8.803	27-Sep-98		Museum Specimen
Arthroleptis bioko	CAS 207283	1	EGBK	Pico Basile	1819	3.628	8.803	27-Sep-98	(+)	Museum Specimen
Arthroleptis bioko	CAS 207284	1	EGBK	Pico Basile	1819	3.628	8.803	28-Sep-98		Museum Specimen
Arthroleptis bioko	CAS 207286	1	EGBK	Pico Basile	1819	3.628	8.803	28-Sep-98		Museum Specimen
Arthroleptis cf. sylvatica	CAS 207926	1	EGBK	Moka Malabo	1414	3.361	8.667	4-Oct-98		Museum Specimen
Arthroleptis poecilonotus	CAS 207917	1	EGBK	Road south of Luba	42	3.468	8.493	14-Oct-98		Museum Specimen
Arthroleptis poecilonotus	CAS 207918	1	EGBK	Pico Basile rd	506	3.709	8.874	17-Oct-98		Museum Specimen
Arthroleptis poecilonotus	CAS 207919	1	EGBK	Pico Basile rd	506	3.709	8.874	17-Oct-98		Museum Specimen
Arthroleptis poecilonotus	CAS 207920	1	EGBK	Pico Basile rd	506	3.709	8.874	17-Oct-98		Museum Specimen
Arthroleptis poecilonotus	CAS 207921	1	EGBK	Pico Basile rd	506	3.709	8.874	17-Oct-98		Museum Specimen
Arthroleptis poecilonotus	CAS 207922	1	EGBK	Pico Basile rd	657	3.696	8.871	17-Oct-98	(+)	Museum Specimen
Arthroleptis poecilonotus	CAS 207923	1	EGBK	Pico Basile rd	657	3.696	8.871	17-Oct-98		Museum Specimen
Arthroleptis poecilonotus	CAS 207924	1	EGBK	Basupu	57	3.711	8.667	18-Oct-98		Museum Specimen
Arthroleptis poecilonotus	CAS 207925	1	EGBK	Basupu	57	3.711	8.667	18-Oct-98		Museum Specimen
Arthroleptis poecilonotus	CAS 207927	1	EGBK	Pico Basile rd	506	3.709	8.874	17-Oct-98		Museum Specimen
Arthroleptis poecilonotus	CAS 207928	1	EGBK	Pico Basile rd	506	3.709	8.874	17-Oct-98		Museum Specimen
Arthroleptis poecilonotus	CAS 207929	1	EGBK	Pico Basile rd	657	3.696	8.871	17-Oct-98		Museum Specimen
Arthroleptis poecilonotus	CAS 207930	1	EGBK	Pico Basile rd	657	3.696	8.871	17-Oct-98		Museum Specimen
Arthroleptis poecilonotus	CAS 207931	1	EGBK	Basupu	57	3.711	8.667	18-Oct-98		Museum Specimen
Arthroleptis variabilis	CAS 207817	1	EGBK	Moka, Río Iladyi	1260	3.332	8.670	1-Oct-98		Museum Specimen
Arthroleptis variabilis	CAS 207818	1	EGBK	Moka, Río Iladyi	1260	3.332	8.670	5-Oct-98		Museum Specimen
Arthroleptis variabilis	CAS 207819	1	EGBK	Moka, Río Iladyi	1260	3.332	8.670	5-Oct-98		Museum Specimen
Arthroleptis variabilis	CAS 207820	1	EGBK	Moka Malabo	1414	3.361	8.667	3-Oct-98		Museum Specimen
Arthroleptis variabilis	CAS 207821	1	EGBK	Moka Malabo	1414	3.361	8.667	3-Oct-98		Museum Specimen
Arthroleptis variabilis	CAS 207822	1	EGBK	Moka Malabo	1414	3.361	8.667	3-Oct-98		Museum Specimen
Arthroleptis variabilis	CAS 207823	1	EGBK	Moka Malabo	1414	3.361	8.667	3-Oct-98		Museum Specimen
Arthroleptis variabilis	CAS 207824	1	EGBK	Moka Malabo	1414	3.361	8.667	4-Oct-98		Museum Specimen
Arthroleptis variabilis	CAS 207825	1	EGBK	Moka Malabo	1414	3.361	8.667	4-Oct-98		Museum Specimen
Arthroleptis variabilis	CAS 207826	1	EGBK	Moka Malabo	1414	3.361	8.667	6-Oct-98		Museum Specimen
Arthroleptis variabilis	CAS 207827	1	EGBK	Moka Malabo	1414	3.362	8.669	4-Oct-98		Museum Specimen
Arthroleptis variabilis	CAS 207828	1	EGBK	Pico Basile rd	548	3.705	8.879	18-Oct-98		Museum Specimen
Hylarana albolabris	CAS 207650	2	EGBK	Luba	2	3.461	8.552	12-Oct-98	(+)	Museum Specimen
Hylarana albolabris	CAS 207651	2	EGBK	Luba	2	3.461	8.552	12-Oct-98	(+)	Museum Specimen
Hylarana albolabris	CAS 207652	2	EGBK	Luba	2	3.461	8.552	12-Oct-98		Museum Specimen
Hylarana albolabris	CAS 207653	2	EGBK	Luba	2	3.461	8.552	12-Oct-98		Museum Specimen
Hylarana albolabris	CAS 207654	2	EGBK	Luba	2	3.461	8.552	12-Oct-98	(+)	Museum Specimen
Hylarana albolabris	CAS 207655	2	EGBK	Luba	2	3.461	8.552	12-Oct-98	(+)	Museum Specimen
Hylarana albolabris	CAS 207656	2	EGBK	Road south of Luba	42	3.468	8.493	14-Oct-98	(+)	Museum Specimen
Hylarana albolabris	CAS 207657	2	EGBK	Road south of Luba	42	3.468	8.493	14-Oct-98		Museum Specimen
Hylarana albolabris	CAS 207658	2	EGBK	Road south of Luba	42	3.468	8.493	14-Oct-98		Museum Specimen
Hylarana albolabris	CAS 207659	2	EGBK	Road south of Luba	42	3.468	8.493	14-Oct-98		Museum Specimen
Hylarana albolabris	CAS 207660	2	EGBK	Road south of Luba	42	3.468	8.493	14-Oct-98		Museum Specimen
Hylarana albolabris	CAS 207689	2	EGBK	Basupu	57	3.711	8.667	18-Oct-98	(+)	Museum Specimen
Hyperolius endjami	CAS 207321	1.5	EGBK	Moka, Río Iladyi	1143	3.325	8.671	1-Oct-98		Museum Specimen
Hyperolius endjami	CAS 207322	1.5	EGBK	Moka, Río Iladyi	1143	3.325	8.671	1-Oct-98		Museum Specimen
Hyperolius endjami	CAS 207323	1.5	EGBK	Moka, Río Iladyi	1143	3.325	8.671	1-Oct-98		Museum Specimen
Hyperolius endjami	CAS 207324	1.5	EGBK	Moka, Río Iladyi	1143	3.325	8.671	1-Oct-98	(+)	Museum Specimen
Hyperolius endjami	CAS 207325	1.5	EGBK	Moka, Río Iladyi	1143	3.325	8.671	1-Oct-98		Museum Specimen
Hyperolius endjami	CAS 207326	1.5	EGBK	Moka, Río Iladyi	1143	3.325	8.671	1-Oct-98		Museum Specimen
Hyperolius endjami	CAS 207327	1.5	EGBK	Moka, Río Iladyi	1143	3.325	8.671	1-Oct-98		Museum Specimen
Hyperolius endjami	CAS 207328	1.5	EGBK	Moka, Río Iladyi	1143	3.325	8.671	1-Oct-98		Museum Specimen
Hyperolius endjami	CAS 207329	1.5	EGBK	Moka, Río Iladyi	1143	3.325	8.671	1-Oct-98		Museum Specimen
Hyperolius endjami	CAS 207330	1.5	EGBK	Moka, Río Iladyi	1143	3.325	8.671	1-Oct-98		Museum Specimen

Species	Cat. No.	Aq. Index	Country	Locality	Elevation (m)	Lat	Long	Collected	*Bd*	Collection Method
Hyperolius endjami	CAS 207331	1.5	EGBK	Moka, Río Iladyi	1143	3.325	8.671	1-Oct-98		Museum Specimen
Hyperolius endjami	CAS 207332	1.5	EGBK	Moka, Río Iladyi	1143	3.325	8.671	1-Oct-98		Museum Specimen
Hyperolius endjami	CAS 207333	1.5	EGBK	Moka, Río Iladyi	1143	3.325	8.671	1-Oct-98		Museum Specimen
Hyperolius endjami	CAS 207334	1.5	EGBK	Moka, Río Iladyi	1143	3.325	8.671	1-Oct-98		Museum Specimen
Hyperolius endjami	CAS 207335	1.5	EGBK	Moka, Río Iladyi	1143	3.325	8.671	1-Oct-98		Museum Specimen
Hyperolius endjami	CAS 207835	1.5	EGBK	Moka Malabo	1414	3.361	8.662	1-Oct-98		Museum Specimen
Hyperolius endjami	CAS 207836	1.5	EGBK	Moka Malabo	1414	3.361	8.662	3-Oct-98		Museum Specimen
Hyperolius endjami	CAS 207837	1.5	EGBK	Moka Malabo	1414	3.361	8.662	3-Oct-98		Museum Specimen
Hyperolius endjami	CAS 207838	1.5	EGBK	Moka Malabo	1414	3.367	8.665	3-Oct-98		Museum Specimen
Hyperolius endjami	CAS 207839	1.5	EGBK	Moka Malabo	1414	3.367	8.665	3-Oct-98		Museum Specimen
Hyperolius endjami	CAS 207840	1.5	EGBK	Moka Malabo	1414	3.367	8.665	3-Oct-98		Museum Specimen
Hyperolius endjami	CAS 207841	1.5	EGBK	Moka Malabo	1414	3.367	8.665	3-Oct-98		Museum Specimen
Hyperolius endjami	CAS 207842	1.5	EGBK	Lago Biao	1860	3.355	8.622	9-Oct-98		Museum Specimen
Hyperolius endjami	CAS 207843	1.5	EGBK	Lago Biao	1860	3.355	8.622	9-Oct-98		Museum Specimen
Hyperolius endjami	CAS 207844	1.5	EGBK	Lago Biao	1860	3.355	8.622	9-Oct-98		Museum Specimen
Hyperolius endjami	CAS 207845	1.5	EGBK	Lago Biao	1860	3.355	8.622	9-Oct-98		Museum Specimen
Hyperolius endjami	CAS 207846	1.5	EGBK	Lago Biao	1860	3.355	8.622	9-Oct-98	(+)	Museum Specimen
Hyperolius endjami	CAS 207847	1.5	EGBK	Lago Biao	1860	3.355	8.622	9-Oct-98		Museum Specimen
Hyperolius endjami	CAS 207848	1.5	EGBK	Lago Biao	1860	3.355	8.622	9-Oct-98		Museum Specimen
Hyperolius endjami	CAS 207849	1.5	EGBK	Lago Biao	1860	3.355	8.622	9-Oct-98	(+)	Museum Specimen
Hyperolius tuberculatus	CAS 207703	1.5	EGBK	Lago Biao	1860	3.355	8.622	9-Oct-98		Museum Specimen
Hyperolius tuberculatus	CAS 207704	1.5	EGBK	Lago Biao	1860	3.355	8.622	9-Oct-98		Museum Specimen
Hyperolius tuberculatus	CAS 207705	1.5	EGBK	Lago Biao	1860	3.355	8.622	9-Oct-98	(+)	Museum Specimen
Hyperolius tuberculatus	CAS 207706	1.5	EGBK	Lago Biao	1860	3.355	8.622	9-Oct-98		Museum Specimen
Hyperolius tuberculatus	CAS 207707	1.5	EGBK	Lago Biao	1860	3.355	8.622	9-Oct-98		Museum Specimen
Hyperolius tuberculatus	CAS 207708	1.5	EGBK	Lago Biao	1860	3.355	8.622	9-Oct-98		Museum Specimen
Hyperolius tuberculatus	CAS 207709	1.5	EGBK	Lago Biao	1860	3.355	8.622	9-Oct-98		Museum Specimen
Hyperolius tuberculatus	CAS 207710	1.5	EGBK	Lago Biao	1860	3.355	8.622	9-Oct-98		Museum Specimen
Hyperolius tuberculatus	CAS 207711	1.5	EGBK	Lago Biao	1860	3.355	8.622	9-Oct-98		Museum Specimen
Hyperolius tuberculatus	CAS 207712	1.5	EGBK	Lago Biao	1860	3.355	8.622	9-Oct-98		Museum Specimen
Hyperolius tuberculatus	CAS 207713	1.5	EGBK	Lago Biao	1860	3.355	8.622	9-Oct-98		Museum Specimen
Hyperolius tuberculatus	CAS 207714	1.5	EGBK	Lago Biao	1860	3.355	8.622	9-Oct-98		Museum Specimen
Hyperolius tuberculatus	CAS 207715	1.5	EGBK	Lago Biao	1860	3.355	8.622	9-Oct-98	(+)	Museum Specimen
Hyperolius tuberculatus	CAS 207716	1.5	EGBK	Lago Biao	1860	3.355	8.622	9-Oct-98		Museum Specimen
Hyperolius tuberculatus	CAS 207717	1.5	EGBK	Lago Biao	1860	3.355	8.622	9-Oct-98		Museum Specimen
Hyperolius tuberculatus	CAS 207718	1.5	EGBK	Lago Biao	1860	3.355	8.622	9-Oct-98		Museum Specimen
Hyperolius tuberculatus	CAS 207719	1.5	EGBK	Lago Biao	1860	3.355	8.622	9-Oct-98		Museum Specimen
Hyperolius tuberculatus	CAS 207720	1.5	EGBK	Lago Biao	1860	3.355	8.622	9-Oct-98		Museum Specimen
Hyperolius tuberculatus	CAS 207721	1.5	EGBK	Lago Biao	1860	3.355	8.622	9-Oct-98		Museum Specimen
Hyperolius tuberculatus	CAS 207722	1.5	EGBK	Lago Biao	1860	3.355	8.622	9-Oct-98		Museum Specimen
Hyperolius tuberculatus	CAS 207723	1.5	EGBK	Lago Biao	1860	3.355	8.622	9-Oct-98		Museum Specimen
Hyperolius tuberculatus	CAS 207724	1.5	EGBK	Lago Biao	1860	3.355	8.622	9-Oct-98		Museum Specimen
Hyperolius tuberculatus	CAS 207725	1.5	EGBK	Lago Biao	1860	3.355	8.622	9-Oct-98		Museum Specimen
Hyperolius tuberculatus	CAS 207726	1.5	EGBK	Lago Biao	1860	3.355	8.622	9-Oct-98		Museum Specimen
Hyperolius tuberculatus	CAS 207727	1.5	EGBK	Lago Biao	1860	3.355	8.622	9-Oct-98		Museum Specimen
Hyperolius tuberculatus	CAS 207728	1.5	EGBK	Lago Biao	1860	3.355	8.622	9-Oct-98		Museum Specimen
Hyperolius tuberculatus	CAS 207729	1.5	EGBK	Lago Biao	1860	3.355	8.622	9-Oct-98	(+)	Museum Specimen
Hyperolius tuberculatus	CAS 207730	1.5	EGBK	Lago Biao	1860	3.355	8.622	9-Oct-98		Museum Specimen
Hyperolius tuberculatus	CAS 207731	1.5	EGBK	Lago Biao	1860	3.355	8.622	9-Oct-98		Museum Specimen
Hyperolius tuberculatus	CAS 207739	1.5	EGBK	Lago Biao	1860	3.355	8.622	9-Oct-98		Museum Specimen
Leptopelis brevirostris	CAS 207830	1	EGBK	Pico Basile rd	571	3.702	8.874	17-Oct-98		Museum Specimen
Leptopelis calcaratus	CAS 207299	1.5	EGBK	Moka, Río Iladyi	1143	3.325	8.671	1-Oct-98		Museum Specimen
Leptopelis calcaratus	CAS 207300	1.5	EGBK	Moka, Río Iladyi	1143	3.325	8.671	1-Oct-98		Museum Specimen
Leptopelis calcaratus	CAS 207301	1.5	EGBK	Moka, Río Iladyi	1143	3.325	8.671	1-Oct-98	(+)	Museum Specimen
Leptopelis calcaratus	CAS 207302	1.5	EGBK	Moka, Río Iladyi	1143	3.325	8.671	1-Oct-98	(+)	Museum Specimen
Leptopelis calcaratus	CAS 207303	1.5	EGBK	Moka, Río Iladyi	1143	3.325	8.671	1-Oct-98		Museum Specimen
Leptopelis calcaratus	CAS 207304	1.5	EGBK	Moka, Río Iladyi	1143	3.325	8.671	1-Oct-98		Museum Specimen
Leptopelis calcaratus	CAS 207305	1.5	EGBK	Moka, Río Iladyi	1143	3.325	8.671	1-Oct-98		Museum Specimen
Leptopelis calcaratus	CAS 207306	1.5	EGBK	Moka, Río Iladyi	1143	3.325	8.671	1-Oct-98		Museum Specimen
Leptopelis calcaratus	CAS 207308	1.5	EGBK	Moka, Río Iladyi	1143	3.325	8.671	1-Oct-98		Museum Specimen
Leptopelis calcaratus	CAS 207309	1.5	EGBK	Moka, Río Iladyi	1143	3.325	8.671	1-Oct-98		Museum Specimen
Leptopelis calcaratus	CAS 207310	1.5	EGBK	Moka, Río Iladyi	1143	3.325	8.671	1-Oct-98	(+)	Museum Specimen
Leptopelis calcaratus	CAS 207311	1.5	EGBK	Moka, Río Iladyi	1143	3.325	8.671	1-Oct-98		Museum Specimen
Leptopelis calcaratus	CAS 207312	1.5	EGBK	Moka, Río Iladyi	1143	3.325	8.671	1-Oct-98	(+)	Museum Specimen
Leptopelis calcaratus	CAS 207313	1.5	EGBK	Moka, Río Iladyi	1143	3.325	8.671	1-Oct-98		Museum Specimen
Leptopelis calcaratus	CAS 207314	1.5	EGBK	Moka, Río Iladyi	1143	3.325	8.671	1-Oct-98		Museum Specimen
Leptopelis calcaratus	CAS 207315	1.5	EGBK	Moka, Río Iladyi	1143	3.325	8.671	5-Oct-98		Museum Specimen
Leptopelis calcaratus	CAS 207316	1.5	EGBK	Moka, Río Iladyi	1143	3.325	8.671	5-Oct-98		Museum Specimen
Leptopelis calcaratus	CAS 207317	1.5	EGBK	Moka, Río Iladyi	1143	3.325	8.671	5-Oct-98		Museum Specimen
Leptopelis calcaratus	CAS 207318	1.5	EGBK	Moka, Río Iladyi	1143	3.325	8.671	6-Oct-98		Museum Specimen
Leptopelis calcaratus	CAS 207319	1.5	EGBK	Moka, Río Iladyi	1143	3.325	8.671	6-Oct-98	(+)	Museum Specimen
Leptopelis calcaratus	CAS 207320	1.5	EGBK	Moka, Río Iladyi	1143	3.325	8.671	6-Oct-98	(+)	Museum Specimen
Leptopelis calcaratus	CAS 207850	1.5	EGBK	Moka Malabo	1414	3.361	8.667	4-Oct-98		Museum Specimen
Leptopelis calcaratus	CAS 207851	1.5	EGBK	Moka Malabo	1414	3.361	8.667	8-Oct-98		Museum Specimen
Leptopelis calcaratus	CAS 207852	1.5	EGBK	Moka, Río Iladyi	1260	3.332	8.670	5-Oct-98		Museum Specimen
Leptopelis cf. aubryi	CAS 207853	1.5	EGBK	Moka Malabo	1264	3.331	8.667	6-Oct-98		Museum Specimen
Leptopelis modestus	CAS 207815	2	EGBK	Moka Malabo	unknown	N/A	N/A	2-Oct-98		Museum Specimen
Leptopelis modestus	CAS 207816	2	EGBK	Moka Malabo	unknown	N/A	N/A	2-Oct-98		Museum Specimen
Nectophryne afra	CAS 207832	1.5	EGBK	Pico Basile rd	657	3.696	8.871	17-Oct-98		Museum Specimen
Nectophryne afra	CAS 207833	1.5	EGBK	Pico Basile rd	657	3.696	8.871	17-Oct-98		Museum Specimen
Nectophryne afra	CAS 207834	1.5	EGBK	Pico Basile rd	657	3.696	8.871	17-Oct-98		Museum Specimen

Species	Cat. No.	Aq. Index	Country	Locality	Elevation (m)	Lat	Long	Collected	*Bd*	Collection Method
Phrynobatrachus africanus	CAS 207780	2	EGBK	Bahia de San Carlos	15	3.483	8.582	13-Oct-98		Museum Specimen
Phrynobatrachus africanus	CAS 207781	2	EGBK	Bahia de San Carlos	15	3.483	8.582	13-Oct-98		Museum Specimen
Phrynobatrachus africanus	CAS 207782	2	EGBK	Bahia de San Carlos	15	3.483	8.582	13-Oct-98		Museum Specimen
Phrynobatrachus auritus	CAS 207933	1.5	EGBK	Moka, Río Iladyi	1260	3.332	8.670	5-Oct-98	(+)	Museum Specimen
Phrynobatrachus auritus	CAS 207934	1.5	EGBK	Moka, Río Iladyi	1264	3.331	8.667	6-Oct-98	(+)	Museum Specimen
Phrynobatrachus auritus	CAS 207935	1.5	EGBK	Moka, Río Iladyi	1264	3.331	8.667	6-Oct-98	(+)	Museum Specimen
Phrynobatrachus auritus	CAS 207936	1.5	EGBK	Moka, Río Iladyi	1264	3.331	8.667	6-Oct-98	(+)	Museum Specimen
Phrynobatrachus auritus	CAS 207939	1.5	EGBK	Moka Malabo	1414	3.367	8.665	9-Oct-98	(+)	Museum Specimen
Phrynobatrachus cornutus	CAS 207800	1.5	EGBK	Bahia de San Carlos	15	3.483	8.582	13-Oct-98		Museum Specimen
Phrynobatrachus cornutus	CAS 207801	1.5	EGBK	Bahia de San Carlos	15	3.483	8.582	13-Oct-98	(+)	Museum Specimen
Phrynobatrachus cornutus	CAS 207802	1.5	EGBK	Bahia de San Carlos	15	3.483	8.582	13-Oct-98		Museum Specimen
Phrynobatrachus cornutus	CAS 207803	1.5	EGBK	Bahia de San Carlos	15	3.483	8.582	13-Oct-98		Museum Specimen
Phrynobatrachus cornutus	CAS 207804	1.5	EGBK	Bahia de San Carlos	15	3.483	8.582	13-Oct-98	(+)	Museum Specimen
Phrynobatrachus cornutus	CAS 207878	1.5	EGBK	Moka, Río Iladyi	1143	3.325	8.671	1-Oct-98		Museum Specimen
Phrynobatrachus cornutus	CAS 207879	1.5	EGBK	Moka, Río Iladyi	1143	3.325	8.671	1-Oct-98		Museum Specimen
Phrynobatrachus cornutus	CAS 207880	1.5	EGBK	Moka, Río Iladyi	1143	3.325	8.671	1-Oct-98		Museum Specimen
Phrynobatrachus cornutus	CAS 207881	1.5	EGBK	Moka, Río Iladyi	1143	3.325	8.671	1-Oct-98	(+)	Museum Specimen
Phrynobatrachus cornutus	CAS 207882	1.5	EGBK	Moka, Río Iladyi	1143	3.325	8.671	1-Oct-98		Museum Specimen
Phrynobatrachus cornutus	CAS 207883	1.5	EGBK	Moka, Río Iladyi	1143	3.325	8.671	1-Oct-98		Museum Specimen
Phrynobatrachus cornutus	CAS 207884	1.5	EGBK	Moka, Río Iladyi	1143	3.325	8.671	1-Oct-98		Museum Specimen
Phrynobatrachus cornutus	CAS 207885	1.5	EGBK	Moka, Río Iladyi	1143	3.325	8.671	1-Oct-98		Museum Specimen
Phrynobatrachus cornutus	CAS 207886	1.5	EGBK	Moka, Río Iladyi	1143	3.325	8.671	1-Oct-98	(+)	Museum Specimen
Phrynobatrachus cornutus	CAS 207887	1.5	EGBK	Moka, Río Iladyi	1143	3.325	8.671	1-Oct-98		Museum Specimen
Phrynobatrachus cornutus	CAS 207888	1.5	EGBK	Moka, Río Iladyi	1264	3.331	8.667	6-Oct-98	(+)	Museum Specimen
Phrynobatrachus cornutus	CAS 207889	1.5	EGBK	Moka, Río Iladyi	1264	3.331	8.667	6-Oct-98	(+)	Museum Specimen
Phrynobatrachus cornutus	CAS 207890	1.5	EGBK	Moka, Río Iladyi	1264	3.331	8.667	6-Oct-98	(+)	Museum Specimen
Phrynobatrachus cornutus	CAS 207891	1.5	EGBK	Moka, Río Iladyi	1264	3.331	8.667	6-Oct-98		Museum Specimen
Phrynobatrachus cornutus	CAS 207892	1.5	EGBK	Moka, Río Iladyi	1264	3.331	8.667	6-Oct-98		Museum Specimen
Phrynobatrachus cornutus	CAS 207893	1.5	EGBK	Moka, Río Iladyi	1264	3.331	8.667	6-Oct-98	(+)	Museum Specimen
Phrynobatrachus cornutus	CAS 207894	1.5	EGBK	Moka, Río Iladyi	1264	3.331	8.667	6-Oct-98	(+)	Museum Specimen
Phrynobatrachus cornutus	CAS 207895	1.5	EGBK	Moka, Río Iladyi	1264	3.331	8.667	6-Oct-98		Museum Specimen
Phrynobatrachus cornutus	CAS 207896	1.5	EGBK	Moka, Río Iladyi	1264	3.331	8.667	6-Oct-98	(+)	Museum Specimen
Phrynobatrachus cornutus	CAS 207897	1.5	EGBK	Moka, Río Iladyi	1264	3.331	8.667	6-Oct-98	(+)	Museum Specimen
Phrynobatrachus cornutus	CAS 207898	1.5	EGBK	Moka, Río Iladyi	1264	3.331	8.667	6-Oct-98	(+)	Museum Specimen
Phrynobatrachus cornutus	CAS 207899	1.5	EGBK	Moka, Río Iladyi	1264	3.331	8.667	6-Oct-98		Museum Specimen
Phrynobatrachus cornutus	CAS 207900	1.5	EGBK	Moka, Río Iladyi	1264	3.331	8.667	6-Oct-98	(+)	Museum Specimen
Phrynobatrachus cornutus	CAS 207901	1.5	EGBK	Moka, Río Iladyi	1264	3.331	8.667	6-Oct-98		Museum Specimen
Phrynobatrachus cornutus	CAS 207915	1.5	EGBK	Pico Basile rd	657	3.696	8.871	17-Oct-98		Museum Specimen
Phrynobatrachus cornutus	CAS 207916	1.5	EGBK	Pico Basile rd	657	3.696	8.871	17-Oct-98	(+)	Museum Specimen
Silurana epitropicalis	CAS 207615	3	EGBK	Basupu	57	3.711	8.667	18-Oct-98		Museum Specimen
Silurana epitropicalis	CAS 207616	3	EGBK	Basupu	57	3.711	8.667	18-Oct-98		Museum Specimen
Silurana epitropicalis	CAS 207617	3	EGBK	Basupu	57	3.711	8.667	18-Oct-98		Museum Specimen
Silurana epitropicalis	CAS 207618	3	EGBK	Basupu	57	3.711	8.667	18-Oct-98		Museum Specimen
Silurana epitropicalis	CAS 207619	3	EGBK	Basupu	57	3.711	8.667	18-Oct-98		Museum Specimen
Silurana epitropicalis	CAS 207752	3	EGBK	Arena Blanca	76	3.520	8.586	14-Oct-98	(+)	Museum Specimen
Silurana epitropicalis	CAS 207753	3	EGBK	Arena Blanca	76	3.520	8.586	14-Oct-98	(+)	Museum Specimen
Silurana epitropicalis	CAS 207755	3	EGBK	Arena Blanca	76	3.520	8.586	14-Oct-98		Museum Specimen
Silurana epitropicalis	CAS 207756	3	EGBK	Arena Blanca	76	3.520	8.586	14-Oct-98		Museum Specimen
Silurana epitropicalis	CAS 207757	3	EGBK	Arena Blanca	76	3.520	8.586	14-Oct-98		Museum Specimen
Silurana epitropicalis	CAS 207758	3	EGBK	Arena Blanca	76	3.520	8.586	14-Oct-98		Museum Specimen
Silurana epitropicalis	CAS 207759	3	EGBK	Arena Blanca	29	3.528	8.579	14-Oct-98	(+)	Museum Specimen
Silurana epitropicalis	CAS 207760	3	EGBK	Arena Blanca	29	3.528	8.579	14-Oct-98		Museum Specimen
Silurana epitropicalis	CAS 207761	3	EGBK	Arena Blanca	29	3.528	8.579	14-Oct-98		Museum Specimen
Silurana epitropicalis	CAS 207762	3	EGBK	Arena Blanca	29	3.528	8.579	14-Oct-98		Museum Specimen
Silurana epitropicalis	CAS 207763	3	EGBK	Arena Blanca	29	3.528	8.579	14-Oct-98		Museum Specimen
Silurana epitropicalis	CAS 207764	3	EGBK	Arena Blanca	29	3.528	8.579	14-Oct-98		Museum Specimen
Xenopus cf. fraseri	CAS 207754	3	EGBK	Arena Blanca	76	3.520	8.586	14-Oct-98		Museum Specimen
Xenopus fraseri	CAS 207741	3	EGBK	Arena Blanca	76	3.520	8.586	14-Oct-98		Museum Specimen
Xenopus fraseri	CAS 207742	3	EGBK	Arena Blanca	76	3.520	8.586	14-Oct-98		Museum Specimen
Xenopus fraseri	CAS 207743	3	EGBK	Arena Blanca	76	3.520	8.586	14-Oct-98		Museum Specimen
Xenopus fraseri	CAS 207744	3	EGBK	Arena Blanca	76	3.520	8.586	14-Oct-98		Museum Specimen
Xenopus fraseri	CAS 207745	3	EGBK	Arena Blanca	76	3.520	8.586	14-Oct-98		Museum Specimen
Xenopus fraseri	CAS 207746	3	EGBK	Arena Blanca	76	3.520	8.586	14-Oct-98		Museum Specimen
Xenopus fraseri	CAS 207747	3	EGBK	Arena Blanca	76	3.520	8.586	14-Oct-98		Museum Specimen
Xenopus fraseri	CAS 207748	3	EGBK	Arena Blanca	76	3.520	8.586	14-Oct-98	(+)	Museum Specimen
Xenopus fraseri	CAS 207749	3	EGBK	Arena Blanca	76	3.520	8.586	14-Oct-98		Museum Specimen
Xenopus fraseri	CAS 207750	3	EGBK	Arena Blanca	76	3.520	8.586	14-Oct-98		Museum Specimen
Xenopus fraseri	CAS 207751	3	EGBK	Arena Blanca	76	3.520	8.586	14-Oct-98	(+)	Museum Specimen
Xenopus fraseri	CAS 207765	3	EGBK	Arena Blanca	29	3.528	8.579	14-Oct-98		Museum Specimen
Xenopus fraseri	CAS 207766	3	EGBK	Arena Blanca	29	3.528	8.579	14-Oct-98		Museum Specimen
Xenopus fraseri	CAS 207767	3	EGBK	Arena Blanca	29	3.528	8.579	14-Oct-98		Museum Specimen
Xenopus fraseri	CAS 207768	3	EGBK	Arena Blanca	29	3.528	8.579	14-Oct-98		Museum Specimen
Xenopus fraseri	CAS 207769	3	EGBK	Arena Blanca	29	3.528	8.579	14-Oct-98		Museum Specimen
Xenopus fraseri	CAS 207770	3	EGBK	Arena Blanca	29	3.528	8.579	14-Oct-98		Museum Specimen
Afrixalus paradorsalis	CUMV 14900	1.5	GAES	Monts de Cristal, Kinguélé	75	0.454	10.278	10-Oct-09		Bell *et al.* 2011
Amietophrynus superciliaris	N/A N/A	2	GAES	Monts de Cristal, Kinguélé	75	0.454	10.278	12-Oct-09		Bell *et al.* 2011
Arthroleptis sp.	MCZ A-139645	1	GAES	Monts de Cristal, Kinguélé	75	0.454	10.278	11-Oct-09		Bell *et al.* 2011
Arthroleptis sp.	NCSM 76804	1	GAES	Monts de Cristal, Kinguélé	75	0.454	10.278	8-Oct-09	(+)	Bell *et al.* 2011
Cardioglossa elegans	MCZ A-139646	2	GAES	Monts de Cristal, Kinguélé	75	0.454	10.278	9-Oct-09	(+)	Bell *et al.* 2011
Cardioglossa leucomystax	MCZ A-139638	2	GAES	Monts de Cristal, Kinguélé	65	0.445	10.282	12-Oct-09		Bell *et al.* 2011
Chiromantis rufescens	MCZ A-139648	1.5	GAES	Monts de Cristal, Kinguélé	75	0.454	10.278	8-Oct-09		Bell *et al.* 2011

Species	Cat. No.	Aq. Index	Country	Locality	Elevation (m)	Lat	Long	Collected	*Bd*	Collection Method
Chiromantis rufescens	MCZ A-139649	1.5	GAES	Monts de Cristal, Kinguélé	75	0.454	10.278	11-Oct-09	(+)	Bell *et al.* 2011
Chiromantis rufescens	NCSM 76844	1.5	GAES	Monts de Cristal, Kinguélé	75	0.454	10.278	11-Oct-09		Bell *et al.* 2011
Conraua crassipes	MCZ A-139652	2.5	GAES	Monts de Cristal, Kinguélé	75	0.454	10.278	10-Oct-09		Bell *et al.* 2011
Hylarana sp.	MCZ A-139637	2	GAES	Monts de Cristal, Kinguélé	75	0.449	10.275	11-Oct-09		Bell *et al.* 2011
Hylarana sp.	MCZ A-139642	2	GAES	Monts de Cristal, Kinguélé	75	0.454	10.278	8-Oct-09		Bell *et al.* 2011
Hylarana sp.	MCZ A-139643	2	GAES	Monts de Cristal, Kinguélé	75	0.454	10.278	11-Oct-09		Bell *et al.* 2011
Hyperolius ocellatus	CUMV 14895	1.5	GAES	Monts de Cristal, Kinguélé	75	0.454	10.278	9-Oct-09	(+)	Bell *et al.* 2011
Hyperolius ocellatus	CUMV 14896	1.5	GAES	Monts de Cristal, Kinguélé	75	0.454	10.278	9-Oct-09		Bell *et al.* 2011
Hyperolius phantasticus	CUMV 14894	1.5	GAES	Monts de Cristal, Kinguélé	75	0.454	10.278	8-Oct-09		Bell *et al.* 2011
Leptopelis aubryi	NCSM 76827	1.5	GAES	Monts de Cristal, Kinguélé	75	0.454	10.278	8-Oct-09		Bell *et al.* 2011
Leptopelis crystallinoron	NCSM 76820	1	GAES	Monts de Cristal, Kinguélé	186	0.450	10.276	11-Oct-09	(+)	Bell *et al.* 2011
Leptopelis rufus	MCZ A-139662	1.5	GAES	Monts de Cristal, Kinguélé	75	0.454	10.278	11-Oct-09		Bell *et al.* 2011
Leptopelis rufus	MCZ A-139664	1.5	GAES	Monts de Cristal, Kinguélé	75	0.454	10.278	9-Oct-09		Bell *et al.* 2011
Leptopelis rufus	MCZ A-139713	1.5	GAES	Monts de Cristal, Kinguélé	115	0.465	10.279	12-Oct-09		Bell *et al.* 2011
Leptopelis rufus	NCSM 76830	1.5	GAES	Monts de Cristal, Kinguélé	75	0.454	10.278	8-Oct-09		Bell *et al.* 2011
Leptopelis zebra	MCZ A-139663	2	GAES	Monts de Cristal, Kinguélé	75	0.454	10.278	8-Oct-09		Bell *et al.* 2011
Nectophryne batesii	NCSM 76798	1.5	GAES	Monts de Cristal, Kinguélé	186	0.450	10.276	9-Oct-09		Bell *et al.* 2011
Petropedetes cf. johnstoni	NCSM 76811	2.5	GAES	Monts de Cristal, Kinguélé	186	0.450	10.276	9-Oct-09		Bell *et al.* 2011
Petropedetes palmipes	MCZ A-139666	2.5	GAES	Monts de Cristal, Kinguélé	75	0.454	10.278	10-Oct-09		Bell *et al.* 2011
Petropedetes palmipes	MCZ A-139667	2.5	GAES	Monts de Cristal, Kinguélé	75	0.454	10.278	10-Oct-09	(+)	Bell *et al.* 2011
Petropedetes palmipes	MCZ A-139674	2.5	GAES	Monts de Cristal, Kinguélé	115	0.454	10.278	12-Oct-09		Bell *et al.* 2011
Petropedetes palmipes	MCZ A-139715	2.5	GAES	Monts de Cristal, Kinguélé	75	0.465	10.279	12-Oct-09		Bell *et al.* 2011
Petropedetes palmipes	NCSM 76813	2.5	GAES	Monts de Cristal, Kinguélé	75	0.454	10.278	10-Oct-09	(+)	Bell *et al.* 2011
Phlyctimantis leonardi	MCZ A-139676	1.5	GAES	Monts de Cristal, Kinguélé	75	0.454	10.278	8-Oct-09		Bell *et al.* 2011
Phlyctimantis leonardi	MCZ A-139684	1.5	GAES	Monts de Cristal, Kinguélé	75	0.454	10.278	11-Oct-09		Bell *et al.* 2011
Phlyctimantis leonardi	MCZ A-139688	1.5	GAES	Monts de Cristal, Kinguélé	75	0.454	10.278	11-Oct-09		Bell *et al.* 2011
Phrynobatrachus auritus	MCZ A-139639	1.5	GAES	Monts de Cristal, Kinguélé	65	0.445	10.282	12-Oct-09	(+)	Bell *et al.* 2011
Ptychadena sp.	MCZ A-139694	2	GAES	Monts de Cristal, Kinguélé	75	0.454	10.278	8-Oct-09	(+)	Bell *et al.* 2011
Scotobleps gabonicus	MCZ A-139706	2.5	GAES	Monts de Cristal, Kinguélé	75	0.454	10.278	10-Oct-09	(+)	Bell *et al.* 2011
Scotobleps gabonicus	MCZ A-139707	2.5	GAES	Monts de Cristal, Kinguélé	75	0.454	10.278	10-Oct-09		Bell *et al.* 2011
Scotobleps gabonicus	NCSM 76817	2.5	GAES	Monts de Cristal, Kinguélé	75	0.454	10.278	10-Oct-09		Bell *et al.* 2011
Silurana paratropicalis	NCSM 76797	3	GAES	Monts de Cristal, Kinguélé	75	0.454	10.278	8-Oct-09		Bell *et al.* 2011
Afrixalus fulvovittatus	CUMV 14934	1.5	GAOI	Ivindo National Park, Ipassa Station	530	0.511	12.803	18-Oct-09		Bell *et al.* 2011
Afrixalus fulvovittatus	CUMV 14936	1.5	GAOI	Ivindo National Park, Ipassa Station	530	0.511	12.803	18-Oct-09	(+)	Bell *et al.* 2011
Afrixalus fulvovittatus	MCZ A-139759	1.5	GAOI	Ivindo National Park, Ipassa Station	530	0.511	12.803	19-Oct-09		Bell *et al.* 2011
Afrixalus fulvovittatus	MCZ A-139760	1.5	GAOI	Ivindo National Park, Ipassa Station	530	0.511	12.803	21-Oct-09		Bell *et al.* 2011
Afrixalus fulvovittatus	NCSM 76838	1.5	GAOI	Ivindo National Park, Ipassa Station	530	0.511	12.803	21-Oct-09		Bell *et al.* 2011
Afrixalus fulvovittatus	CUMV 14918	1.5	GAOI	Ivindo National Park, Ipassa Station	530	0.511	12.803	17-Oct-09		Bell *et al.* 2011
Afrixalus paradorsalis	CUMV 14920	1.5	GAOI	Ivindo National Park, Ipassa Station	530	0.511	12.803	17-Oct-09		Bell *et al.* 2011
Afrixalus paradorsalis	CUMV 14928	1.5	GAOI	Ivindo National Park, Ipassa Station	530	0.511	12.803	18-Oct-09	(+)	Bell *et al.* 2011
Afrixalus paradorsalis	CUMV 14929	1.5	GAOI	Ivindo National Park, Ipassa Station	530	0.511	12.803	18-Oct-09		Bell *et al.* 2011
Afrixalus paradorsalis	CUMV 15024	1.5	GAOI	Ivindo National Park, Ipassa Station	530	0.511	12.803	12-Oct-10		Field-caught specimen
Afrixalus paradorsalis	MCZ A-139761	1.5	GAOI	Ivindo National Park, Ipassa Station	530	0.511	12.803	17-Oct-09		Bell *et al.* 2011
Amietophrynus camerunensis	MCZ A-139745	1.5	GAOI	Ivindo National Park, Ipassa Station	500	0.516	12.795	21-Oct-09		Bell *et al.* 2011
Amietophrynus camerunensis	NCSM 76800	1.5	GAOI	Ivindo National Park, Ipassa Station	530	0.511	12.803	18-Oct-09		Bell *et al.* 2011
Amietophrynus camerunensis	NCSM 78927	1.5	GAOI	Ivindo, Rougier Gabon Forestry Concession	188	-0.226	12.298	15-Oct-11	(+)	Field-caught specimen
Amietophrynus camerunensis	NCSM 78928	1.5	GAOI	Ivindo, Rougier Gabon Forestry Concession	188	-0.226	12.298	15-Oct-11	(+)	Field-caught specimen
Amietophrynus camerunensis	NCSM 78929	1.5	GAOI	Ivindo, Rougier Gabon Forestry Concession	188	-0.226	12.298	15-Oct-11		Field-caught specimen
Amietophrynus camerunensis	NCSM 78930	1.5	GAOI	Ivindo, Rougier Gabon Forestry Concession	188	-0.226	12.298	15-Oct-11		Field-caught specimen
Amietophrynus camerunensis	NCSM 78931	1.5	GAOI	Ivindo, Rougier Gabon Forestry Concession	188	-0.226	12.298	15-Oct-11		Field-caught specimen
Amietophrynus camerunensis	NCSM 78932	1.5	GAOI	Ivindo, Rougier Gabon Forestry Concession	188	-0.226	12.298	15-Oct-11		Field-caught specimen
Amietophrynus camerunensis	NCSM 78933	1.5	GAOI	Ivindo, Rougier Gabon Forestry Concession	209	-0.215	12.300	15-Oct-11		Field-caught specimen
Amietophrynus camerunensis	NCSM 78934	1.5	GAOI	Ivindo, Rougier Gabon Forestry Concession	209	-0.215	12.300	15-Oct-11		Field-caught specimen
Amietophrynus camerunensis	NCSM 78935	1.5	GAOI	Ivindo, Rougier Gabon Forestry Concession	209	-0.215	12.300	15-Oct-11		Field-caught specimen
Amietophrynus camerunensis	NCSM 78936	1.5	GAOI	Ivindo, Rougier Gabon Forestry Concession	209	-0.215	12.300	15-Oct-11		Field-caught specimen
Amietophrynus camerunensis	NCSM 78939	1.5	GAOI	Ivindo, Rougier Gabon Forestry Concession	249	-0.039	12.291	18-Oct-11	(+)	Field-caught specimen
Amietophrynus gracilipes	MCZ A-139716	2	GAOI	Ivindo National Park, Ipassa Station	500	0.500	12.802	22-Oct-09		Bell *et al.* 2011
Amietophrynus gracilipes	NCSM 76801	2	GAOI	Ivindo National Park, Ipassa Station	480	0.504	12.794	20-Oct-09		Bell *et al.* 2011
Amietophrynus gracilipes	NCSM 78920	2	GAOI	Ivindo, Rougier Gabon Forestry Concession	276	-0.043	12.298	19-Oct-11		Field-caught specimen
Arthroleptis adelphus	MCZ A-139775	1	GAOI	Ivindo National Park, Ipassa Station	530	0.511	12.803	22-Oct-09		Bell *et al.* 2011
Arthroleptis sp.	MCZ A-139754	1	GAOI	Ivindo National Park, Ipassa Station	500	0.516	12.795	22-Oct-09		Bell *et al.* 2011
Arthroleptis sp.	MCZ A-139776	1	GAOI	Ivindo National Park, Ipassa Station	530	0.511	12.803	18-Oct-09	(+)	Bell *et al.* 2011
Arthroleptis sp.	MCZ A-139777	1	GAOI	Ivindo National Park, Ipassa Station	530	0.511	12.803	18-Oct-09		Bell *et al.* 2011
Arthroleptis sp.	MCZ A-139778	1	GAOI	Ivindo National Park, Ipassa Station	530	0.511	12.803	19-Oct-09		Bell *et al.* 2011
Arthroleptis sp.	MCZ A-139779	1	GAOI	Ivindo National Park, Ipassa Station	530	0.511	12.803	19-Oct-09		Bell *et al.* 2011
Arthroleptis sp.	MCZ A-139780	1	GAOI	Ivindo National Park, Ipassa Station	530	0.511	12.803	20-Oct-09		Bell *et al.* 2011
Arthroleptis sp.	NCSM 76805	1	GAOI	Ivindo National Park, Ipassa Station	530	0.511	12.803	18-Oct-09		Bell *et al.* 2011
Arthroleptis sp.	NCSM 76806	1	GAOI	Ivindo National Park, Ipassa Station	530	0.511	12.803	20-Oct-09		Bell *et al.* 2011
Arthroleptis sp.	NCSM 77647	1	GAOI	Ivindo National Park, Ipassa Station	530	0.511	12.803	16-Oct-10	(+)	Field-caught specimen
Arthroleptis variabilis	NCSM 78889	1	GAOI	Ivindo, Rougier Gabon Forestry Concession	188	-0.226	12.298	15-Oct-11		Field-caught specimen
Arthroleptis variabilis	NCSM 78890	1	GAOI	Ivindo, Rougier Gabon Forestry Concession	249	-0.039	12.291	17-Oct-11		Field-caught specimen
Arthroleptis variabilis	NCSM 78891	1	GAOI	Ivindo, Rougier Gabon Forestry Concession	249	-0.039	12.291	17-Oct-11		Field-caught specimen
Arthroleptis variabilis	NCSM 78892	1	GAOI	Ivindo, Rougier Gabon Forestry Concession	249	-0.039	12.291	17-Oct-11		Field-caught specimen
Arthroleptis variabilis	NCSM 78894	1	GAOI	Ivindo, Rougier Gabon Forestry Concession	249	-0.039	12.291	18-Oct-11		Field-caught specimen
Arthroleptis variabilis	NCSM 78895	1	GAOI	Ivindo, Rougier Gabon Forestry Concession	276	-0.043	12.298	18-Oct-11		Field-caught specimen
Astylosternus batesi	MCZ A-139783	2.5	GAOI	Ivindo National Park, Ipassa Station	530	0.511	12.803	17-Oct-09		Bell *et al.* 2011
Astylosternus batesi	NCSM 76810	2.5	GAOI	Ivindo National Park, Ipassa Station	480	0.504	12.794	20-Oct-09		Bell *et al.* 2011
Cardioglossa leucomystax	NCSM 78887	2	GAOI	Ivindo, Rougier Gabon Forestry Concession	270	-0.056	12.313	19-Oct-11		Field-caught specimen
Chiromantis rufescens	MCZ A-139720	1.5	GAOI	Ivindo National Park, Ipassa Station	500	0.500	12.802	22-Oct-09		Bell *et al.* 2011
Chiromantis rufescens	MCZ A-139784	1.5	GAOI	Ivindo National Park, Ipassa Station	530	0.511	12.803	17-Oct-09		Bell *et al.* 2011
Chiromantis rufescens	MCZ A-139786	1.5	GAOI	Ivindo National Park, Ipassa Station	530	0.511	12.803	18-Oct-09		Bell *et al.* 2011

Species	Cat. No.	Aq. Index	Country	Locality	Elevation (m)	Lat	Long	Collected	*Bd*	Collection Method
Chiromantis rufescens	MCZ A-139787	1.5	GAOI	Ivindo National Park, Ipassa Station	530	0.511	12.803	18-Oct-09	(+)	Bell *et al.* 2011
Chiromantis rufescens	MCZ A-139790	1.5	GAOI	Ivindo National Park, Ipassa Station	530	0.511	12.803	21-Oct-09	(+)	Bell *et al.* 2011
Conraua crassipes	MCZ A-139721	2.5	GAOI	Ivindo National Park, Ipassa Station	480	0.504	12.794	20-Oct-09		Bell *et al.* 2011
Conraua crassipes	MCZ A-139722	2.5	GAOI	Ivindo National Park, Ipassa Station	480	0.504	12.794	20-Oct-09		Bell *et al.* 2011
Conraua crassipes	MCZ A-139723	2.5	GAOI	Ivindo National Park, Ipassa Station	480	0.504	12.794	20-Oct-09		Bell *et al.* 2011
Conraua crassipes	MCZ A-139724	2.5	GAOI	Ivindo National Park, Ipassa Station	480	0.504	12.794	20-Oct-09		Bell *et al.* 2011
Conraua crassipes	MCZ A-139725	2.5	GAOI	Ivindo National Park, Ipassa Station	480	0.504	12.794	20-Oct-09		Bell *et al.* 2011
Conraua crassipes	MCZ A-139726	2.5	GAOI	Ivindo National Park, Ipassa Station	480	0.504	12.794	20-Oct-09	(+)	Bell *et al.* 2011
Conraua crassipes	NCSM 76816	2.5	GAOI	Ivindo National Park, Ipassa Station	480	0.500	12.802	22-Oct-09		Bell *et al.* 2011
Hylarana sp.	MCZ A-139717	2	GAOI	Ivindo National Park, Ipassa Station	500	0.500	12.802	22-Oct-09	(+)	Bell *et al.* 2011
Hylarana sp.	MCZ A-139718	2	GAOI	Ivindo National Park, Ipassa Station	500	0.500	12.802	22-Oct-09		Bell *et al.* 2011
Hylarana sp.	MCZ A-139746	2	GAOI	Ivindo National Park, Ipassa Station	500	0.516	12.795	19-Oct-09	(+)	Bell *et al.* 2011
Hylarana sp.	MCZ A-139747	2	GAOI	Ivindo National Park, Ipassa Station	500	0.516	12.795	19-Oct-09		Bell *et al.* 2011
Hylarana sp.	MCZ A-139748	2	GAOI	Ivindo National Park, Ipassa Station	500	0.516	12.795	21-Oct-09		Bell *et al.* 2011
Hylarana sp.	MCZ A-139749	2	GAOI	Ivindo National Park, Ipassa Station	500	0.516	12.795	22-Oct-09		Bell *et al.* 2011
Hylarana sp.	MCZ A-139752	2	GAOI	Ivindo National Park, Ipassa Station	500	0.516	12.795	19-Oct-09		Bell *et al.* 2011
Hylarana sp.	MCZ A-139773	2	GAOI	Ivindo National Park, Ipassa Station	530	0.511	12.803	17-Oct-09	(+)	Bell *et al.* 2011
Hylarana sp.	MCZ A-139774	2	GAOI	Ivindo National Park, Ipassa Station	530	0.511	12.803	18-Oct-09		Bell *et al.* 2011
Hylarana sp.	NCSM 76851	2	GAOI	Ivindo National Park, Ipassa Station	500	0.516	12.795	19-Oct-09		Bell *et al.* 2011
Hylarana sp.	NCSM 77629	2	GAOI	Ivindo National Park, Ipassa Station	530	0.511	12.803	11-Oct-10		Field-caught specimen
Hylarana sp.	NCSM 77631	2	GAOI	Ivindo National Park, Ipassa Station	480	0.500	12.802	11-Oct-10	(+)	Field-caught specimen
Hylarana sp.	NCSM 77632	2	GAOI	Ivindo National Park, Ipassa Station	540	0.506	12.796	12-Oct-10	(+)	Field-caught specimen
Hyperolius bolifambae	CUMV 14942	1.5	GAOI	Ivindo National Park, Ipassa Station	480	0.500	12.802	22-Oct-09		Bell *et al.* 2011
Hyperolius bolifambae	CUMV 14943	1.5	GAOI	Ivindo National Park, Ipassa Station	480	0.500	12.802	22-Oct-09		Bell *et al.* 2011
Hyperolius bolifambae	CUMV 14944	1.5	GAOI	Ivindo National Park, Ipassa Station	480	0.500	12.802	22-Oct-09		Bell *et al.* 2011
Hyperolius cinnamomeoventris	CUMV 14948	1.5	GAOI	Ivindo National Park, Ipassa Station	480	0.500	12.802	22-Oct-09		Bell *et al.* 2011
Hyperolius cinnamomeoventris	CUMV 14949	1.5	GAOI	Ivindo National Park, Ipassa Station	480	0.500	12.802	22-Oct-09		Bell *et al.* 2011
Hyperolius cinnamomeoventris	CUMV 14950	1.5	GAOI	Ivindo National Park, Ipassa Station	480	0.500	12.802	22-Oct-09		Bell *et al.* 2011
Hyperolius cinnamomeoventris	CUMV 14952	1.5	GAOI	Ivindo National Park, Ipassa Station	530	0.511	12.803	21-Oct-09		Bell *et al.* 2011
Hyperolius cinnamomeoventris	CUMV 14953	1.5	GAOI	Ivindo National Park, Ipassa Station	530	0.511	12.803	21-Oct-09		Bell *et al.* 2011
Hyperolius cinnamomeoventris	CUMV 14954	1.5	GAOI	Ivindo National Park, Ipassa Station	530	0.511	12.803	21-Oct-09		Bell *et al.* 2011
Hyperolius cinnamomeoventris	CUMV 14955	1.5	GAOI	Ivindo National Park, Ipassa Station	530	0.511	12.803	21-Oct-09		Bell *et al.* 2011
Hyperolius kuligae	CUMV 14930	1.5	GAOI	Ivindo National Park, Ipassa Station	530	0.511	12.803	18-Oct-09		Bell *et al.* 2011
Hyperolius ocellatus	CUMV 14891	1.5	GAOI	Ivindo National Park, Ipassa Station	480	0.500	12.802	22-Oct-09		Bell *et al.* 2011
Hyperolius ocellatus	CUMV 14892	1.5	GAOI	Ivindo National Park, Ipassa Station	480	0.500	12.802	22-Oct-09	(+)	Bell *et al.* 2011
Hyperolius ocellatus	CUMV 14939	1.5	GAOI	Ivindo National Park, Ipassa Station	480	0.504	12.794	20-Oct-09	(+)	Bell *et al.* 2011
Hyperolius ocellatus	CUMV 14956	1.5	GAOI	Ivindo National Park, Ipassa Station	500	0.516	12.795	19-Oct-09		Bell *et al.* 2011
Hyperolius ocellatus	CUMV 14957	1.5	GAOI	Ivindo National Park, Ipassa Station	500	0.516	12.795	19-Oct-09		Bell *et al.* 2011
Hyperolius ocellatus	CUMV 14958	1.5	GAOI	Ivindo National Park, Ipassa Station	500	0.516	12.795	19-Oct-09		Bell *et al.* 2011
Hyperolius ocellatus	CUMV 14959	1.5	GAOI	Ivindo National Park, Ipassa Station	500	0.516	12.795	19-Oct-09		Bell *et al.* 2011
Hyperolius ocellatus	CUMV 14960	1.5	GAOI	Ivindo National Park, Ipassa Station	500	0.516	12.795	19-Oct-09	(+)	Bell *et al.* 2011
Hyperolius ocellatus	CUMV 14989	1.5	GAOI	Ivindo National Park, Ipassa Station	530	0.511	12.803	16-Oct-10		Field-caught specimen
Hyperolius ocellatus	CUMV 14993	1.5	GAOI	Ivindo National Park, Kongou Camp	460	0.294	12.566	13-Oct-10	(+)	Field-caught specimen
Hyperolius ocellatus	CUMV 14997	1.5	GAOI	Ivindo National Park, Kongou Camp	460	0.294	12.566	13-Oct-10	(+)	Field-caught specimen
Hyperolius ocellatus	CUMV 15536	1.5	GAOI	Ivindo, Rougier Gabon Forestry Concession	276	-0.043	12.298	18-Oct-11		Field-caught specimen
Hyperolius tuberculatus	CUMV 15578	1.5	GAOI	Ivindo, Rougier Gabon Forestry Concession	210	-0.196	12.196	23-Oct-11		Field-caught specimen
Leptopelis aubryi	NCSM 76829	1.5	GAOI	Ivindo National Park, Ipassa Station	530	0.511	12.803	17-Oct-09	(+)	Bell *et al.* 2011
Leptopelis aubryi	NCSM 76835	1.5	GAOI	Ivindo National Park, Ipassa Station	480	0.504	12.794	20-Oct-09		Bell *et al.* 2011
Leptopelis aubryioides	NCSM 76831	1.5	GAOI	Ivindo National Park, Ipassa Station	530	0.511	12.803	17-Oct-09		Bell *et al.* 2011
Leptopelis aubryioides	NCSM 77689	1.5	GAOI	Ivindo National Park, Ipassa Station	480	0.500	12.802	17-Oct-10		Field-caught specimen
Leptopelis aubryioides	NCSM 77705	1.5	GAOI	Ivindo National Park, Ipassa Station	530	0.502	12.800	17-Oct-10		Field-caught specimen
Leptopelis boulengeri	MCZ A-139798	1.5	GAOI	Ivindo National Park, Ipassa Station	530	0.511	12.803	17-Oct-09		Bell *et al.* 2011
Leptopelis boulengeri	NCSM 77675	1.5	GAOI	Ivindo National Park, Ipassa Station	540	0.506	12.796	12-Oct-10	(+)	Field-caught specimen
Leptopelis brevirostris	MCZ A-139794	1	GAOI	Ivindo National Park, Ipassa Station	530	0.511	12.803	19-Oct-09		Bell *et al.* 2011
Leptopelis brevirostris	MCZ A-139796	1	GAOI	Ivindo National Park, Ipassa Station	530	0.511	12.803	22-Oct-09		Bell *et al.* 2011
Leptopelis brevirostris	NCSM 76826	1	GAOI	Ivindo National Park, Ipassa Station	530	0.511	12.803	20-Oct-09		Bell *et al.* 2011
Leptopelis calcaratus	NCSM 77691	1.5	GAOI	Ivindo National Park, Ipassa Station	480	0.500	12.802	11-Oct-10		Field-caught specimen
Leptopelis calcaratus	NCSM 77693	1.5	GAOI	Ivindo National Park, Kongou Camp	475	0.293	12.574	14-Oct-10		Field-caught specimen
Leptopelis calcaratus	NCSM 77697	1.5	GAOI	Ivindo National Park, Ipassa Station	480	0.500	12.802	17-Oct-10		Field-caught specimen
Leptopelis notatus	MCZ A-139795	1.5	GAOI	Ivindo National Park, Ipassa Station	530	0.511	12.803	20-Oct-09	(+)	Bell *et al.* 2011
Leptopelis notatus	NCSM 77682	1.5	GAOI	Ivindo National Park, Ipassa Station	540	0.506	12.796	12-Oct-10	(+)	Field-caught specimen
Leptopelis notatus	NCSM 77683	1.5	GAOI	Ivindo National Park, Ipassa Station	530	0.511	12.803	16-Oct-10		Field-caught specimen
Leptopelis notatus	NCSM 77684	1.5	GAOI	Ivindo National Park, Ipassa Station	530	0.511	12.803	16-Oct-10		Field-caught specimen
Leptopelis notatus	NCSM 78957	1.5	GAOI	Ivindo, Rougier Gabon Forestry Concession	270	-0.056	12.313	19-Oct-11		Field-caught specimen
Leptopelis ocellatus	MCZ A-139729	1.5	GAOI	Ivindo National Park, Ipassa Station	480	0.504	12.794	20-Oct-09	(+)	Bell *et al.* 2011
Leptopelis ocellatus	MCZ A-139730	1.5	GAOI	Ivindo National Park, Ipassa Station	480	0.504	12.794	22-Oct-09		Bell *et al.* 2011
Leptopelis ocellatus	MCZ A-139731	1.5	GAOI	Ivindo National Park, Ipassa Station	480	0.504	12.794	22-Oct-09	(+)	Bell *et al.* 2011
Leptopelis ocellatus	MCZ A-139732	1.5	GAOI	Ivindo National Park, Ipassa Station	500	0.500	12.802	22-Oct-09		Bell *et al.* 2011
Leptopelis ocellatus	MCZ A-139733	1.5	GAOI	Ivindo National Park, Ipassa Station	500	0.500	12.802	22-Oct-09		Bell *et al.* 2011
Leptopelis ocellatus	MCZ A-139734	1.5	GAOI	Ivindo National Park, Ipassa Station	500	0.500	12.802	22-Oct-09		Bell *et al.* 2011
Leptopelis ocellatus	MCZ A-139735	1.5	GAOI	Ivindo National Park, Ipassa Station	500	0.500	12.802	22-Oct-09	(+)	Bell *et al.* 2011
Leptopelis ocellatus	MCZ A-139755	1.5	GAOI	Ivindo National Park, Ipassa Station	500	0.516	12.795	21-Oct-09		Bell *et al.* 2011
Leptopelis ocellatus	MCZ A-139793	1.5	GAOI	Ivindo National Park, Ipassa Station	530	0.511	12.803	18-Oct-09		Bell *et al.* 2011
Leptopelis ocellatus	NCSM 76822	1.5	GAOI	Ivindo National Park, Ipassa Station	530	0.511	12.803	17-Oct-09		Bell *et al.* 2011
Leptopelis ocellatus	NCSM 76823	1.5	GAOI	Ivindo National Park, Ipassa Station	530	0.511	12.803	18-Oct-09		Bell *et al.* 2011
Leptopelis ocellatus	NCSM 76824	1.5	GAOI	Ivindo National Park, Ipassa Station	500	0.516	12.795	21-Oct-09		Bell *et al.* 2011
Leptopelis ocellatus	NCSM 77678	1.5	GAOI	Ivindo National Park, Kongou Camp	490	0.290	12.573	13-Oct-10		Field-caught specimen
Leptopelis ocellatus	NCSM 78956	1.5	GAOI	Ivindo, Rougier Gabon Forestry Concession	188	-0.226	12.298	16-Oct-11	(+)	Field-caught specimen
Leptopelis ocellatus	NCSM 78958	1.5	GAOI	Ivindo, Rougier Gabon Forestry Concession	224	-0.189	12.185	20-Oct-11		Field-caught specimen
Leptopelis ocellatus	NCSM 78959	1.5	GAOI	Ivindo, Rougier Gabon Forestry Concession	224	-0.189	12.185	20-Oct-11	(+)	Field-caught specimen
Leptopelis ocellatus	NCSM 78960	1.5	GAOI	Ivindo, Rougier Gabon Forestry Concession	224	-0.189	12.185	20-Oct-11		Field-caught specimen

Species	Cat. No.	Aq. Index	Country	Locality	Elevation (m)	Lat	Long	Collected	*Bd*	Collection Method
Leptopelis ocellatus	NCSM 78961	1.5	GAOI	Ivindo, Rougier Gabon Forestry Concession	224	-0.189	12.185	20-Oct-11	(+)	Field-caught specimen
Leptopelis ocellatus	NCSM 78962	1.5	GAOI	Ivindo, Rougier Gabon Forestry Concession	224	-0.189	12.185	20-Oct-11		Field-caught specimen
Leptopelis ocellatus	NCSM 78963	1.5	GAOI	Ivindo, Rougier Gabon Forestry Concession	224	-0.189	12.185	20-Oct-11		Field-caught specimen
Leptopelis ocellatus	NCSM 78964	1.5	GAOI	Ivindo, Rougier Gabon Forestry Concession	224	-0.189	12.185	20-Oct-11		Field-caught specimen
Leptopelis ocellatus	NCSM 78965	1.5	GAOI	Ivindo, Rougier Gabon Forestry Concession	224	-0.189	12.185	20-Oct-11		Field-caught specimen
Leptopelis ocellatus	NCSM 78966	1.5	GAOI	Ivindo, Rougier Gabon Forestry Concession	224	-0.189	12.185	20-Oct-11	(+)	Field-caught specimen
Leptopelis rufus	NCSM 77702	1.5	GAOI	Ivindo National Park, Kongou Camp	475	0.293	12.574	14-Oct-10		Field-caught specimen
Leptopelis sp.	MCZ A-139740	1.5	GAOI	Ivindo National Park, Ipassa Station	500	0.500	12.802	22-Oct-09		Bell *et al.* 2011
Leptopelis sp.	MCZ A-139741	1.5	GAOI	Ivindo National Park, Ipassa Station	500	0.500	12.802	22-Oct-09	(+)	Bell *et al.* 2011
Leptopelis sp.	MCZ A-139756	1.5	GAOI	Ivindo National Park, Ipassa Station	500	0.516	12.795	19-Oct-09		Bell *et al.* 2011
Leptopelis sp.	MCZ A-139757	1.5	GAOI	Ivindo National Park, Ipassa Station	500	0.516	12.795	21-Oct-09		Bell *et al.* 2011
Leptopelis sp.	MCZ A-139799	1.5	GAOI	Ivindo National Park, Ipassa Station	530	0.511	12.803	17-Oct-09	(+)	Bell *et al.* 2011
Leptopelis sp.	MCZ A-139800	1.5	GAOI	Ivindo National Park, Ipassa Station	530	0.511	12.803	18-Oct-09		Bell *et al.* 2011
Nectophryne afra	NCSM 77617	1.5	GAOI	Ivindo National Park, Ipassa Station	545	0.511	12.800	11-Oct-10		Field-caught specimen
Nectophryne batesii	MCZ A-139758	1.5	GAOI	Ivindo National Park, Ipassa Station	500	0.516	12.795	19-Oct-09		Bell *et al.* 2011
Nectophryne batesii	MCZ A-139801	1.5	GAOI	Ivindo National Park, Ipassa Station	530	0.511	12.803	20-Oct-09		Bell *et al.* 2011
Opisthothylax immaculatus	CUMV 14938	1.5	GAOI	Ivindo National Park, Ipassa Station	480	0.504	12.794	20-Oct-09		Bell *et al.* 2011
Opisthothylax immaculatus	CUMV 14941	1.5	GAOI	Ivindo National Park, Ipassa Station	500	0.516	12.795	19-Oct-09		Bell *et al.* 2011
Phrynobatrachus africanus	MCZ A-139728	2	GAOI	Ivindo National Park, Ipassa Station	480	0.504	12.794	20-Oct-09		Bell *et al.* 2011
Phrynobatrachus sp.	NCSM 77623	1.5	GAOI	Ivindo National Park, Ipassa Station	530	0.511	12.803	11-Oct-10		Field-caught specimen
Ptychadena sp.	MCZ A-139742	2	GAOI	Ivindo National Park, Ipassa Station	500	0.500	12.802	22-Oct-09		Bell *et al.* 2011
Ptychadena sp.	MCZ A-139743	2	GAOI	Ivindo National Park, Ipassa Station	500	0.500	12.802	22-Oct-09		Bell *et al.* 2011
Ptychadena sp.	MCZ A-139744	2	GAOI	Ivindo National Park, Ipassa Station	480	0.504	12.794	20-Oct-09	(+)	Bell *et al.* 2011
Ptychadena sp.	MCZ A-139803	2	GAOI	Ivindo National Park, Ipassa Station	530	0.511	12.803	18-Oct-09		Bell *et al.* 2011
Ptychadena sp.	MCZ A-139804	2	GAOI	Ivindo National Park, Ipassa Station	530	0.511	12.803	18-Oct-09		Bell *et al.* 2011
Ptychadena sp.	MCZ A-140271	2	GAOI	Ivindo National Park, Ipassa Station	530	0.511	12.803	16-Oct-10		Field-caught specimen
Ptychadena sp.	MCZ A-140279	2	GAOI	Ivindo National Park, Kongou Camp	490	0.290	12.573	15-Oct-10		Field-caught specimen
Ptychadena sp.	NCSM 77652	2	GAOI	Ivindo National Park, Kongou Camp	490	0.290	12.573	13-Oct-10	(+)	Field-caught specimen
Ptychadena sp.	NCSM 78946	2	GAOI	Ivindo, Rougier Gabon Forestry Concession	249	-0.039	12.291	18-Oct-11	(+)	Field-caught specimen
Ptychadena sp.	NCSM 78947	2	GAOI	Ivindo, Rougier Gabon Forestry Concession	249	-0.039	12.291	18-Oct-11		Field-caught specimen
Ptychadena sp.	NCSM 78948	2	GAOI	Ivindo, Rougier Gabon Forestry Concession	249	-0.039	12.291	18-Oct-11		Field-caught specimen
Ptychadena sp.	NCSM 78950	2	GAOI	Ivindo, Rougier Gabon Forestry Concession	249	-0.039	12.291	18-Oct-11		Field-caught specimen
Ptychadena sp.	NCSM 78951	2	GAOI	Ivindo, Rougier Gabon Forestry Concession	249	-0.039	12.291	18-Oct-11		Field-caught specimen
Ptychadena sp.	NCSM 78952	2	GAOI	Ivindo, Rougier Gabon Forestry Concession	249	-0.039	12.291	18-Oct-11	(+)	Field-caught specimen
Xenopus parafraseri	NCSM 78874	3	GAOI	Ivindo, Rougier Gabon Forestry Concession	276	-0.043	12.298	21-Oct-11		Field-caught specimen
Hylarana sp.	MCZ A-140296	2	GAOM	Loango National Park, Rembo Rabi River	17	-1.892	9.568	25-Oct-10		Field-caught specimen
Hylarana sp.	MCZ A-140301	2	GAOM	Loango National Park, Rembo Rabi River	9	-1.889	9.569	26-Oct-10		Field-caught specimen
Hylarana sp.	NCSM 77640	2	GAOM	Loango National Park, Rembo Rabi River	11	-1.893	9.567	25-Oct-10		Field-caught specimen
Hyperolius sp.	CUMV 14990	1.5	GAOM	Loango National Park, Rembo Rabi River	7	-1.891	9.568	25-Oct-10		Field-caught specimen
Hyperolius sp.	CUMV 15098	1.5	GAOM	Iguéla	16	-1.814	9.356	24-Oct-10	(+)	Field-caught specimen
Leptopelis aubryi	MCZ A-140286	1.5	GAOM	Iguéla	28	-1.823	9.380	24-Oct-10		Field-caught specimen
Leptopelis aubryi	MCZ A-140288	1.5	GAOM	Iguéla	14	-1.910	9.316	23-Oct-10		Field-caught specimen
Leptopelis aubryi	NCSM 77663	1.5	GAOM	Iguéla	14	-1.910	9.316	23-Oct-10		Field-caught specimen
Leptopelis aubryi	NCSM 77664	1.5	GAOM	Iguéla	14	-1.910	9.316	23-Oct-10		Field-caught specimen
Leptopelis aubryi	NCSM 77671	1.5	GAOM	Iguéla	28	-1.823	9.380	24-Oct-10		Field-caught specimen
Phrynobatrachus sp.	MCZ A-140295	1.5	GAOM	Loango National Park, Rembo Rabi River	11	-1.893	9.567	25-Oct-10		Field-caught specimen
Phrynobatrachus sp.	NCSM 77624	1.5	GAOM	Loango National Park, Rembo Rabi River	17	-1.892	9.568	25-Oct-10	(+)	Field-caught specimen
Phrynobatrachus sp.	NCSM 77625	1.5	GAOM	Loango National Park, Rembo Rabi River	17	-1.892	9.568	26-Oct-10	(+)	Field-caught specimen
Phrynobatrachus sp.	NCSM 77646	1.5	GAOM	Loango National Park, Rembo Rabi River	17	-1.891	9.579	26-Oct-10	(+)	Field-caught specimen
Ptychadena sp.	NCSM 77650	2	GAOM	Iguéla	16	-1.814	9.356	24-Oct-10	(+)	Field-caught specimen
Ptychadena sp.	NCSM 77657	2	GAOM	Loango National Park, Rembo Rabi River	11	-1.893	9.567	25-Oct-10		Field-caught specimen
Cardioglossa leucomystax	NCSM 76808	2	GAWN	Monts de Cristal, Tchimbélé	455	0.626	10.397	13-Oct-09		Bell *et al.* 2011
Hylarana sp.	MCZ A-139815	2	GAWN	Monts de Cristal, Tchimbélé	455	0.626	10.397	13-Oct-09	(+)	Bell *et al.* 2011
Hyperolius tuberculatus	CUMV 14908	1.5	GAWN	Monts de Cristal, Tchimbélé	530	0.621	10.408	14-Oct-09		Bell *et al.* 2011
Hyperolius tuberculatus	CUMV 14910	1.5	GAWN	Monts de Cristal, Tchimbélé	530	0.621	10.408	14-Oct-09		Bell *et al.* 2011
Hyperolius tuberculatus	CUMV 14911	1.5	GAWN	Monts de Cristal, Tchimbélé	530	0.621	10.408	14-Oct-09	(+)	Bell *et al.* 2011
Hyperolius tuberculatus	CUMV 14912	1.5	GAWN	Monts de Cristal, Tchimbélé	530	0.621	10.408	14-Oct-09		Bell *et al.* 2011
Leptopelis crystallinoron	MCZ A-139810	1	GAWN	Monts de Cristal, Tchimbélé	455	0.626	10.397	14-Oct-09		Bell *et al.* 2011
Leptopelis crystallinoron	MCZ A-139811	1	GAWN	Monts de Cristal, Tchimbélé	455	0.626	10.397	14-Oct-09	(+)	Bell *et al.* 2011
Leptopelis crystallinoron	MCZ A-139813	1	GAWN	Monts de Cristal, Tchimbélé	565	0.623	10.403	14-Oct-09		Bell *et al.* 2011
Leptopelis crystallinoron	NCSM 76821	1	GAWN	Monts de Cristal, Tchimbélé	455	0.626	10.397	14-Oct-09		Bell *et al.* 2011
Leptopelis sp.	MCZ A-139814	1.5	GAWN	Monts de Cristal, Tchimbélé	565	0.623	10.403	13-Oct-09	(+)	Bell *et al.* 2011
Petropedetes cf. johnstoni	MCZ A-139816	2.5	GAWN	Monts de Cristal, Tchimbélé	565	0.626	10.397	13-Oct-09	(+)	Bell *et al.* 2011
Petropedetes cf. johnstoni	NCSM 76812	2.5	GAWN	Monts de Cristal, Tchimbélé	455	0.626	10.397	14-Oct-09		Bell *et al.* 2011
Phlyctimantis leonardi	MCZ A-139806	1.5	GAWN	Monts de Cristal, Tchimbélé	530	0.621	10.408	14-Oct-09		Bell *et al.* 2011
Phrynobatrachus auritus	MCZ A-139812	1.5	GAWN	Monts de Cristal, Tchimbélé	455	0.626	10.397	14-Oct-09	(+)	Bell *et al.* 2011
Phrynobatrachus auritus	NCSM 76803	1.5	GAWN	Monts de Cristal, Tchimbélé	455	0.626	10.397	14-Oct-09	(+)	Bell *et al.* 2011
Ptychadena sp.	MCZ A-139807	2	GAWN	Monts de Cristal, Tchimbélé	530	0.621	10.408	14-Oct-09		Bell *et al.* 2011
Ptychadena sp.	MCZ A-139808	2	GAWN	Monts de Cristal, Tchimbélé	530	0.621	10.408	14-Oct-09		Bell *et al.* 2011
Ptychadena sp.	NCSM 76855	2	GAWN	Monts de Cristal, Tchimbélé	455	0.626	10.397	14-Oct-09		Bell *et al.* 2011
Scotobleps gabonicus	MCZ A-139809	2.5	GAWN	Monts de Cristal, Tchimbélé	530	0.621	10.408	14-Oct-09	(+)	Bell *et al.* 2011
Scotobleps gabonicus	MCZ A-139817	2.5	GAWN	Monts de Cristal, Tchimbélé	455	0.626	10.397	13-Oct-09		Bell *et al.* 2011
Scotobleps gabonicus	MCZ A-139818	2.5	GAWN	Monts de Cristal, Tchimbélé	455	0.626	10.397	13-Oct-09	(+)	Bell *et al.* 2011
Scotobleps gabonicus	NCSM 76818	2.5	GAWN	Monts de Cristal, Tchimbélé	455	0.626	10.397	13-Oct-09		Bell *et al.* 2011
Scotobleps gabonicus	NCSM 76819	2.5	GAWN	Monts de Cristal, Tchimbélé	455	0.626	10.397	13-Oct-09		Bell *et al.* 2011
Trichobatrachus robustus	MCZ A-139819	2	GAWN	Monts de Cristal, Tchimbélé	455	0.626	10.397	13-Oct-09		Bell *et al.* 2011
Hyperolius molleri	CAS 219125	1.5	STPR	Chada, Agua Doutor	178	1.652	7.416	19-Apr-01		Museum Specimen
Hyperolius molleri	CAS 219126	1.5	STPR	Chada, Agua Doutor	178	1.652	7.416	19-Apr-01		Museum Specimen
Hyperolius molleri	CAS 219127	1.5	STPR	Chada, Agua Doutor	178	1.652	7.416	19-Apr-01		Museum Specimen
Hyperolius molleri	CAS 219128	1.5	STPR	Chada, Agua Doutor	178	1.652	7.416	19-Apr-01		Museum Specimen
Hyperolius molleri	CAS 219129	1.5	STPR	Chada, Agua Doutor	178	1.652	7.416	19-Apr-01		Museum Specimen

Species	Cat. No.	Aq. Index	Country	Locality	Elevation (m)	Lat	Long	Collected	*Bd*	Collection Method
Hyperolius molleri	CAS 219130	1.5	STPR	Chada, Agua Doutor	178	1.652	7.416	19-Apr-01		Museum Specimen
Hyperolius molleri	CAS 219131	1.5	STPR	Chada, Agua Doutor	178	1.652	7.416	19-Apr-01		Museum Specimen
Hyperolius molleri	CAS 219132	1.5	STPR	Chada, Agua Doutor	178	1.652	7.416	19-Apr-01		Museum Specimen
Hyperolius molleri	CAS 219133	1.5	STPR	Chada, Agua Doutor	178	1.652	7.416	19-Apr-01		Museum Specimen
Hyperolius molleri	CAS 219134	1.5	STPR	Chada, Agua Doutor	178	1.652	7.416	19-Apr-01	(+)	Museum Specimen
Hyperolius molleri	CAS 219136	1.5	STPR	Chada, Agua Doutor	178	1.652	7.416	19-Apr-01		Museum Specimen
Hyperolius molleri	CAS 219137	1.5	STPR	Chada, Agua Doutor	178	1.652	7.416	19-Apr-01		Museum Specimen
Hyperolius molleri	CAS 219138	1.5	STPR	Chada, Agua Doutor	178	1.652	7.416	19-Apr-01		Museum Specimen
Hyperolius molleri	CAS 219139	1.5	STPR	Chada, Agua Doutor	178	1.652	7.416	19-Apr-01		Museum Specimen
Hyperolius molleri	CAS 219140	1.5	STPR	Chada, Agua Doutor	178	1.652	7.416	19-Apr-01		Museum Specimen
Hyperolius molleri	CAS 219141	1.5	STPR	Chada, Agua Doutor	178	1.652	7.416	19-Apr-01		Museum Specimen
Hyperolius molleri	CAS 219142	1.5	STPR	Chada, Agua Doutor	178	1.652	7.416	19-Apr-01		Museum Specimen
Hyperolius molleri	CAS 219148	1.5	STPR	Airport	182	1.669	7.413	20-Apr-01		Museum Specimen
Hyperolius molleri	CAS 219192	1.5	STPR	Alto Conceiçao and Pincate Road	181	1.644	7.398	21-Apr-01	(+)	Museum Specimen
Hyperolius molleri	CAS 219193	1.5	STPR	Alto Conceiçao and Pincate Road	181	1.644	7.398	21-Apr-01	(+)	Museum Specimen
Hyperolius molleri	CAS 219194	1.5	STPR	Alto Conceiçao and Pincate Road	181	1.644	7.398	21-Apr-01		Museum Specimen
Hyperolius molleri	CAS 219195	1.5	STPR	Alto Conceiçao and Pincate Road	181	1.644	7.398	21-Apr-01		Museum Specimen
Hyperolius molleri	CAS 219196	1.5	STPR	Alto Conceiçao and Pincate Road	181	1.644	7.398	21-Apr-01		Museum Specimen
Hyperolius molleri	CAS 219197	1.5	STPR	Alto Conceiçao and Pincate Road	181	1.644	7.398	21-Apr-01		Museum Specimen
Hyperolius molleri	CAS 219203	1.5	STPR	Baie das Agulahs	17	1.601	7.353	22-Apr-01		Museum Specimen
Hyperolius molleri	CAS 219204	1.5	STPR	Baie das Agulahs	17	1.601	7.353	22-Apr-01		Museum Specimen
Hyperolius molleri	CAS 219205	1.5	STPR	Baie das Agulahs	17	1.601	7.353	22-Apr-01		Museum Specimen
Hyperolius molleri	CAS 219206	1.5	STPR	Baie das Agulahs	17	1.601	7.353	22-Apr-01		Museum Specimen
Hyperolius molleri	CAS 219207	1.5	STPR	Baie das Agulahs	17	1.601	7.353	22-Apr-01		Museum Specimen
Hyperolius molleri	CAS 233422	1.5	STPR	Sundy	176	1.661	7.394	11-May-06		Museum Specimen
Leptopelis palmatus	CAS 219177	2	STPR	Sundy	182	1.666	7.381	21-Apr-01		Museum Specimen
Leptopelis palmatus	CAS 219351	2	STPR	Rio Papagaio	176	1.639	7.396	15-May-01		Museum Specimen
Leptopelis palmatus	CAS 219370	2	STPR	Pico do Príncipe, base camp	618	1.589	7.380	17-May-01		Museum Specimen
Leptopelis palmatus	CAS 219371	2	STPR	Pico do Príncipe, base camp	618	1.589	7.380	17-May-01		Museum Specimen
Leptopelis palmatus	CAS 219372	2	STPR	Pico do Príncipe, base camp	618	1.589	7.380	17-May-01		Museum Specimen
Leptopelis palmatus	CAS 219373	2	STPR	Pico do Príncipe, base camp	618	1.589	7.380	17-May-01	(+)	Museum Specimen
Leptopelis palmatus	CAS 219374	2	STPR	Pico do Príncipe, base camp	618	1.589	7.380	17-May-01		Museum Specimen
Leptopelis palmatus	CAS 219375	2	STPR	Pico do Príncipe, base camp	618	1.589	7.380	17-May-01		Museum Specimen
Leptopelis palmatus	CAS 219376	2	STPR	Pico do Príncipe, base camp	618	1.589	7.380	17-May-01		Museum Specimen
Leptopelis palmatus	CAS 219377	2	STPR	Pico do Príncipe, base camp	618	1.589	7.380	17-May-01		Museum Specimen
Leptopelis palmatus	CAS 219378	2	STPR	Pico do Príncipe, base camp	618	1.589	7.380	17-May-01		Museum Specimen
Leptopelis palmatus	CAS 219379	2	STPR	Pico do Príncipe, base camp	618	1.589	7.380	17-May-01		Museum Specimen
Leptopelis palmatus	CAS 219380	2	STPR	Pico do Príncipe, base camp	618	1.589	7.380	17-May-01		Museum Specimen
Leptopelis palmatus	CAS 219381	2	STPR	Pico do Príncipe, base camp	618	1.589	7.380	17-May-01		Museum Specimen
Leptopelis palmatus	CAS 219382	2	STPR	Pico do Príncipe, base camp	618	1.589	7.380	17-May-01		Museum Specimen
Leptopelis palmatus	CAS 219383	2	STPR	Pico do Príncipe, base camp	618	1.589	7.380	17-May-01		Museum Specimen
Leptopelis palmatus	CAS 219400	2	STPR	Pico do Príncipe, base camp	618	1.589	7.380	18-May-01		Museum Specimen
Leptopelis palmatus	CAS 219401	2	STPR	Pico do Príncipe, base camp	618	1.589	7.380	18-May-01		Museum Specimen
Leptopelis palmatus	CAS 233428	2	STPR	Pico Papagaio	180	1.622	7.385	12-May-06	(+)	Museum Specimen
Leptopelis palmatus	CAS 233434	2	STPR	Pico do Príncipe, base camp	620	1.588	7.381	12-May-06		Museum Specimen
Leptopelis palmatus	CAS 233435	2	STPR	Pico do Príncipe, base camp	620	1.588	7.381	12-May-06	(+)	Museum Specimen
Leptopelis palmatus	CAS 233436	2	STPR	Pico do Príncipe, base camp	620	1.588	7.381	12-May-06		Museum Specimen
Leptopelis palmatus	CAS 233437	2	STPR	Pico do Príncipe, base camp	620	1.588	7.381	12-May-06	(+)	Museum Specimen
Leptopelis palmatus	CAS 233438	2	STPR	Pico do Príncipe, base camp	620	1.588	7.381	12-May-06	(+)	Museum Specimen
Leptopelis palmatus	CAS 233439	2	STPR	Pico do Príncipe, base camp	620	1.588	7.381	12-May-06		Museum Specimen
Leptopelis palmatus	CAS 233440	2	STPR	Pico do Príncipe, base camp	620	1.588	7.381	12-May-06		Museum Specimen
Leptopelis palmatus	CAS 233441	2	STPR	Pico do Príncipe, base camp	620	1.588	7.381	12-May-06		Museum Specimen
Leptopelis palmatus	CAS 233442	2	STPR	Pico do Príncipe, base camp	620	1.588	7.381	12-May-06		Museum Specimen
Leptopelis palmatus	CAS 233443	2	STPR	Pico do Príncipe, base camp	620	1.588	7.381	12-May-06		Museum Specimen
Leptopelis palmatus	CAS 238874	2	STPR	Pico Papagaio	250	1.619	7.392	21-Apr-08		Museum Specimen
Leptopelis palmatus	CAS 238881	2	STPR	Pico Papagaio	250	1.618	7.392	28-Apr-08		Museum Specimen
Leptopelis palmatus	CAS 251573	2	STPR	Pico do Mesa	357	1.583	7.356	28-May-12		Museum Specimen
Phrynobatrachus dispar	CAS 219085	1.5	STPR	Chada, Agua Doutor	178	1.652	7.416	19-Apr-01		Museum Specimen
Phrynobatrachus dispar	CAS 219087	1.5	STPR	Chada, Agua Doutor	178	1.652	7.416	19-Apr-01		Museum Specimen
Phrynobatrachus dispar	CAS 219088	1.5	STPR	Chada, Agua Doutor	178	1.652	7.416	19-Apr-01		Museum Specimen
Phrynobatrachus dispar	CAS 219089	1.5	STPR	Chada, Agua Doutor	178	1.652	7.416	19-Apr-01		Museum Specimen
Phrynobatrachus dispar	CAS 219090	1.5	STPR	Chada, Agua Doutor	178	1.652	7.416	19-Apr-01		Museum Specimen
Phrynobatrachus dispar	CAS 219091	1.5	STPR	Chada, Agua Doutor	178	1.652	7.416	19-Apr-01		Museum Specimen
Phrynobatrachus dispar	CAS 219092	1.5	STPR	Chada, Agua Doutor	178	1.652	7.416	19-Apr-01		Museum Specimen
Phrynobatrachus dispar	CAS 219094	1.5	STPR	Chada, Agua Doutor	178	1.652	7.416	19-Apr-01		Museum Specimen
Phrynobatrachus dispar	CAS 219095	1.5	STPR	Chada, Agua Doutor	178	1.652	7.416	19-Apr-01		Museum Specimen
Phrynobatrachus dispar	CAS 219096	1.5	STPR	Chada, Agua Doutor	178	1.652	7.416	19-Apr-01		Museum Specimen
Phrynobatrachus dispar	CAS 219097	1.5	STPR	Chada, Agua Doutor	178	1.652	7.416	19-Apr-01		Museum Specimen
Phrynobatrachus dispar	CAS 219143	1.5	STPR	Airport	182	1.669	7.413	20-Apr-01		Museum Specimen
Phrynobatrachus dispar	CAS 219144	1.5	STPR	Airport	182	1.669	7.413	20-Apr-01	(+)	Museum Specimen
Phrynobatrachus dispar	CAS 219145	1.5	STPR	Airport	182	1.669	7.413	20-Apr-01		Museum Specimen
Phrynobatrachus dispar	CAS 219146	1.5	STPR	Airport	182	1.669	7.413	20-Apr-01		Museum Specimen
Phrynobatrachus dispar	CAS 219147	1.5	STPR	Airport	182	1.669	7.413	20-Apr-01		Museum Specimen
Phrynobatrachus dispar	CAS 219201	1.5	STPR	Baie das Agulahs	17	1.601	7.353	22-Apr-01		Museum Specimen
Phrynobatrachus dispar	CAS 219202	1.5	STPR	Baie das Agulahs	17	1.601	7.353	22-Apr-01		Museum Specimen
Phrynobatrachus dispar	CAS 219345	1.5	STPR	Alto Conceiçao and Pincate Road	193	1.647	7.399	14-May-01		Museum Specimen
Phrynobatrachus dispar	CAS 219346	1.5	STPR	Alto Conceiçao and Pincate Road	180	1.639	7.397	14-May-01		Museum Specimen
Phrynobatrachus dispar	CAS 219352	1.5	STPR	Rio Papagaio	176	1.639	7.396	15-May-01		Museum Specimen
Phrynobatrachus dispar	CAS 219353	1.5	STPR	Rio Papagaio	176	1.639	7.396	15-May-01		Museum Specimen
Phrynobatrachus dispar	CAS 219354	1.5	STPR	Rio Papagaio	176	1.639	7.396	15-May-01	(+)	Museum Specimen
Phrynobatrachus dispar	CAS 219355	1.5	STPR	Rio Papagaio	176	1.639	7.396	15-May-01		Museum Specimen

Species	Cat. No.	Aq. Index	Country	Locality	Elevation (m)	Lat	Long	Collected	*Bd*	Collection Method
Phrynobatrachus dispar	CAS 219356	1.5	STPR	Rio Papagaio	176	1.639	7.396	15-May-01		Museum Specimen
Phrynobatrachus dispar	CAS 219363	1.5	STPR	Santo Antonio	28	1.637	7.414	16-May-01		Museum Specimen
Phrynobatrachus dispar	CAS 219385	1.5	STPR	Pico do Príncipe, base camp	618	1.589	7.380	17-May-01		Museum Specimen
Phrynobatrachus dispar	CAS 219386	1.5	STPR	Pico do Príncipe, base camp	618	1.589	7.380	17-May-01		Museum Specimen
Phrynobatrachus dispar	CAS 219387	1.5	STPR	Pico do Príncipe, base camp	618	1.589	7.380	17-May-01		Museum Specimen
Phrynobatrachus dispar	CAS 219388	1.5	STPR	Pico do Príncipe, base camp	618	1.589	7.380	17-May-01		Museum Specimen
Phrynobatrachus dispar	CAS 219390	1.5	STPR	Pico do Príncipe, base camp	618	1.589	7.380	17-May-01		Museum Specimen
Phrynobatrachus dispar	CAS 219391	1.5	STPR	Pico do Príncipe, base camp	618	1.589	7.380	17-May-01		Museum Specimen
Phrynobatrachus dispar	CAS 219392	1.5	STPR	Pico do Príncipe, base camp	618	1.589	7.380	17-May-01		Museum Specimen
Phrynobatrachus dispar	CAS 219393	1.5	STPR	Pico do Príncipe, base camp	357	1.583	7.383	18-May-01		Museum Specimen
Phrynobatrachus dispar	CAS 233419	1.5	STPR	Sundy	178	1.659	7.395	11-May-06		Museum Specimen
Phrynobatrachus dispar	CAS 233423	1.5	STPR	Sundy	176	1.661	7.394	11-May-06		Museum Specimen
Phrynobatrachus dispar	CAS 233527	1.5	STPR	Rio Papagaio	45	1.630	7.418	10-May-06		Museum Specimen
Phrynobatrachus dispar	CAS 233528	1.5	STPR	Rio Papagaio	45	1.630	7.418	10-May-06		Museum Specimen
Phrynobatrachus dispar	CAS 233529	1.5	STPR	Rio Papagaio	41	1.623	7.416	10-May-06		Museum Specimen
Phrynobatrachus dispar	CAS 233530	1.5	STPR	Rio Papagaio	41	1.623	7.416	10-May-06		Museum Specimen
Phrynobatrachus dispar	CAS 233531	1.5	STPR	Rio Papagaio	41	1.623	7.416	10-May-06		Museum Specimen
Phrynobatrachus dispar	CAS 233534	1.5	STPR	Rio Papagaio	41	1.628	7.417	10-May-06	(+)	Museum Specimen
Phrynobatrachus dispar	CAS 233535	1.5	STPR	Pico do Príncipe, base camp	620	1.588	7.381	12-May-06		Museum Specimen
Phrynobatrachus dispar	CAS 233536	1.5	STPR	Pico do Príncipe, base camp	620	1.588	7.381	12-May-06		Museum Specimen
Phrynobatrachus dispar	CAS 233537	1.5	STPR	Pico do Príncipe, base camp	620	1.588	7.381	12-May-06		Museum Specimen
Phrynobatrachus dispar	CAS 233538	1.5	STPR	Pico do Príncipe, base camp	620	1.588	7.381	12-May-06		Museum Specimen
Phrynobatrachus dispar	CAS 233539	1.5	STPR	Pico do Príncipe, base camp	620	1.588	7.381	12-May-06		Museum Specimen
Phrynobatrachus dispar	CAS 233540	1.5	STPR	Pico do Príncipe, base camp	620	1.588	7.381	12-May-06		Museum Specimen
Phrynobatrachus dispar	CAS 233541	1.5	STPR	Pico do Príncipe, base camp	620	1.588	7.381	12-May-06		Museum Specimen
Phrynobatrachus dispar	CAS 233542	1.5	STPR	Pico do Príncipe, base camp	620	1.588	7.381	12-May-06		Museum Specimen
Phrynobatrachus dispar	CAS 233543	1.5	STPR	Pico do Príncipe, base camp	620	1.588	7.381	12-May-06		Museum Specimen
Phrynobatrachus dispar	CAS 233544	1.5	STPR	Pico do Príncipe, base camp	620	1.588	7.381	12-May-06		Museum Specimen
Phrynobatrachus dispar	CAS 233545	1.5	STPR	Pico do Príncipe, base camp	620	1.588	7.381	12-May-06		Museum Specimen
Phrynobatrachus dispar	CAS 233546	1.5	STPR	Pico do Príncipe, base camp	620	1.588	7.381	12-May-06		Museum Specimen
Phrynobatrachus dispar	CAS 233547	1.5	STPR	Pico do Príncipe, base camp	620	1.588	7.381	12-May-06	(+)	Museum Specimen
Phrynobatrachus dispar	CAS 233548	1.5	STPR	Pico do Príncipe, base camp	620	1.588	7.381	12-May-06		Museum Specimen
Phrynobatrachus dispar	CAS 233549	1.5	STPR	Pico do Príncipe, base camp	620	1.588	7.381	12-May-06		Museum Specimen
Phrynobatrachus dispar	CAS 233550	1.5	STPR	Pico do Príncipe, base camp	620	1.588	7.381	12-May-06	(+)	Museum Specimen
Phrynobatrachus dispar	CAS 233551	1.5	STPR	Pico do Príncipe, base camp	620	1.588	7.381	12-May-06		Museum Specimen
Phrynobatrachus dispar	CAS 233552	1.5	STPR	Pico do Príncipe, base camp	620	1.588	7.381	12-May-06		Museum Specimen
Phrynobatrachus dispar	CAS 233553	1.5	STPR	Pico do Príncipe, base camp	620	1.588	7.381	12-May-06		Museum Specimen
Phrynobatrachus dispar	CAS 233554	1.5	STPR	Pico do Príncipe, base camp	620	1.588	7.381	12-May-06		Museum Specimen
Phrynobatrachus dispar	CAS 233555	1.5	STPR	Pico do Príncipe, base camp	620	1.588	7.381	12-May-06	(+)	Museum Specimen
Phrynobatrachus dispar	CAS 233556	1.5	STPR	Pico do Príncipe, base camp	620	1.588	7.381	12-May-06	(+)	Museum Specimen
Phrynobatrachus dispar	CAS 233563	1.5	STPR	Pico do Príncipe, base camp	620	1.588	7.381	12-May-06		Museum Specimen
Phrynobatrachus dispar	CAS 233569	1.5	STPR	Pico do Príncipe	950	1.580	7.384	13-May-06	(+)	Museum Specimen
Phrynobatrachus dispar	CAS 238892	1.5	STPR	Bom Bom	24	1.689	7.403	30-Apr-08		Museum Specimen
Phrynobatrachus dispar	CAS 238893	1.5	STPR	Bom Bom	24	1.689	7.403	30-Apr-08		Museum Specimen
Hyperolius molleri	CAS 218848	1.5	STST	Caxueira	54	0.298	6.730	5-Apr-01		Museum Specimen
Hyperolius molleri	CAS 218849	1.5	STST	Caxueira	54	0.298	6.730	5-Apr-01		Museum Specimen
Hyperolius molleri	CAS 218850	1.5	STST	Caxueira	54	0.298	6.730	5-Apr-01		Museum Specimen
Hyperolius molleri	CAS 218851	1.5	STST	Caxueira	54	0.298	6.730	5-Apr-01		Museum Specimen
Hyperolius molleri	CAS 218852	1.5	STST	Caxueira	54	0.298	6.730	5-Apr-01		Museum Specimen
Hyperolius molleri	CAS 218853	1.5	STST	Caxueira	54	0.298	6.730	5-Apr-01		Museum Specimen
Hyperolius molleri	CAS 218854	1.5	STST	Caxueira	54	0.298	6.730	5-Apr-01		Museum Specimen
Hyperolius molleri	CAS 218855	1.5	STST	Caxueira	54	0.298	6.730	5-Apr-01		Museum Specimen
Hyperolius molleri	CAS 218856	1.5	STST	Caxueira	54	0.298	6.730	5-Apr-01		Museum Specimen
Hyperolius molleri	CAS 218857	1.5	STST	Caxueira	54	0.298	6.730	5-Apr-01	(+)	Museum Specimen
Hyperolius molleri	CAS 218858	1.5	STST	Caxueira	54	0.298	6.730	5-Apr-01	(+)	Museum Specimen
Hyperolius molleri	CAS 218859	1.5	STST	Caxueira	54	0.298	6.730	5-Apr-01		Museum Specimen
Hyperolius molleri	CAS 218860	1.5	STST	Caxueira	54	0.298	6.730	5-Apr-01		Museum Specimen
Hyperolius molleri	CAS 218862	1.5	STST	Caxueira	49	0.300	6.732	5-Apr-01		Museum Specimen
Hyperolius molleri	CAS 218863	1.5	STST	Caxueira	49	0.300	6.732	5-Apr-01		Museum Specimen
Hyperolius molleri	CAS 218864	1.5	STST	Caxueira	49	0.300	6.732	5-Apr-01		Museum Specimen
Hyperolius molleri	CAS 218865	1.5	STST	Caxueira	49	0.300	6.732	5-Apr-01	(+)	Museum Specimen
Hyperolius molleri	CAS 218866	1.5	STST	Caxueira	49	0.300	6.732	5-Apr-01	(+)	Museum Specimen
Hyperolius molleri	CAS 218867	1.5	STST	Caxueira	49	0.300	6.732	5-Apr-01	(+)	Museum Specimen
Hyperolius molleri	CAS 218868	1.5	STST	Caxueira	49	0.300	6.732	5-Apr-01		Museum Specimen
Hyperolius molleri	CAS 218869	1.5	STST	Caxueira	49	0.300	6.732	5-Apr-01		Museum Specimen
Hyperolius molleri	CAS 218870	1.5	STST	Caxueira	49	0.300	6.732	5-Apr-01		Museum Specimen
Hyperolius molleri	CAS 218871	1.5	STST	Caxueira	49	0.300	6.732	5-Apr-01		Museum Specimen
Hyperolius molleri	CAS 218872	1.5	STST	Caxueira	49	0.300	6.732	5-Apr-01		Museum Specimen
Hyperolius molleri	CAS 218873	1.5	STST	Caxueira	49	0.300	6.732	5-Apr-01		Museum Specimen
Hyperolius molleri	CAS 218874	1.5	STST	Caxueira	49	0.300	6.732	5-Apr-01		Museum Specimen
Hyperolius molleri	CAS 218875	1.5	STST	Caxueira	49	0.300	6.732	5-Apr-01		Museum Specimen
Hyperolius molleri	CAS 218876	1.5	STST	Caxueira	49	0.300	6.732	5-Apr-01		Museum Specimen
Hyperolius molleri	CAS 218877	1.5	STST	Caxueira	49	0.300	6.732	5-Apr-01	(+)	Museum Specimen
Hyperolius molleri	CAS 218975	1.5	STST	Java	592	0.261	6.651	2-Apr-01		Museum Specimen
Hyperolius molleri	CAS 218976	1.5	STST	Java	592	0.261	6.651	11-Apr-01		Museum Specimen
Hyperolius molleri	CAS 218977	1.5	STST	Java	592	0.261	6.651	11-Apr-01		Museum Specimen
Hyperolius molleri	CAS 218978	1.5	STST	Java	592	0.261	6.651	11-Apr-01		Museum Specimen
Hyperolius molleri	CAS 218979	1.5	STST	Java	592	0.261	6.651	11-Apr-01		Museum Specimen
Hyperolius molleri	CAS 218980	1.5	STST	Java	592	0.261	6.651	11-Apr-01		Museum Specimen
Hyperolius molleri	CAS 218981	1.5	STST	Java	592	0.261	6.651	11-Apr-01		Museum Specimen
Hyperolius molleri	CAS 218982	1.5	STST	Java	592	0.261	6.651	11-Apr-01		Museum Specimen

Species	Cat. No.	Aq. Index	Country	Locality	Elevation (m)	Lat	Long	Collected	*Bd*	Collection Method
Hyperolius molleri	CAS 218983	1.5	STST	Java	592	0.261	6.651	11-Apr-01		Museum Specimen
Hyperolius molleri	CAS 218984	1.5	STST	Java	592	0.261	6.651	11-Apr-01		Museum Specimen
Hyperolius molleri	CAS 218985	1.5	STST	Java	592	0.261	6.651	11-Apr-01		Museum Specimen
Hyperolius molleri	CAS 218986	1.5	STST	Java	592	0.261	6.651	11-Apr-01		Museum Specimen
Hyperolius molleri	CAS 218987	1.5	STST	Java	592	0.261	6.651	11-Apr-01		Museum Specimen
Hyperolius molleri	CAS 218988	1.5	STST	Java	592	0.261	6.651	11-Apr-01		Museum Specimen
Hyperolius molleri	CAS 218989	1.5	STST	Java	592	0.261	6.651	11-Apr-01		Museum Specimen
Hyperolius molleri	CAS 218990	1.5	STST	Java	592	0.261	6.651	11-Apr-01		Museum Specimen
Hyperolius molleri	CAS 218991	1.5	STST	Java	592	0.261	6.651	11-Apr-01		Museum Specimen
Hyperolius molleri	CAS 218992	1.5	STST	Java	592	0.261	6.651	11-Apr-01		Museum Specimen
Hyperolius molleri	CAS 218993	1.5	STST	Java	592	0.261	6.651	11-Apr-01		Museum Specimen
Hyperolius molleri	CAS 219047	1.5	STST	Lagoa Amélia	1444	0.282	6.591	14-Apr-01	(+)	Museum Specimen
Hyperolius molleri	CAS 219048	1.5	STST	Lagoa Amélia	1444	0.282	6.591	14-Apr-01		Museum Specimen
Hyperolius molleri	CAS 219049	1.5	STST	Lagoa Amélia	1444	0.282	6.591	14-Apr-01		Museum Specimen
Hyperolius molleri	CAS 219050	1.5	STST	Lagoa Amélia	1444	0.282	6.591	14-Apr-01		Museum Specimen
Hyperolius molleri	CAS 219054	1.5	STST	Bom Sucesso	1254	0.288	6.603	14-Apr-01		Museum Specimen
Hyperolius molleri	CAS 219055	1.5	STST	Bom Sucesso	1254	0.288	6.603	14-Apr-01		Museum Specimen
Hyperolius molleri	CAS 219056	1.5	STST	Bom Sucesso	1254	0.288	6.603	14-Apr-01		Museum Specimen
Hyperolius molleri	CAS 219057	1.5	STST	Bom Sucesso	1254	0.288	6.603	14-Apr-01		Museum Specimen
Hyperolius molleri	CAS 219058	1.5	STST	Bom Sucesso	1254	0.288	6.603	14-Apr-01	(+)	Museum Specimen
Hyperolius molleri	CAS 219059	1.5	STST	Bom Sucesso	1254	0.288	6.603	14-Apr-01	(+)	Museum Specimen
Hyperolius molleri	CAS 219068	1.5	STST	Praia Melão	15	0.319	6.738	17-Apr-01		Museum Specimen
Hyperolius molleri	CAS 219069	1.5	STST	Praia Melão	15	0.319	6.738	17-Apr-01	(+)	Museum Specimen
Hyperolius molleri	CAS 251579	1.5	STST	Bem Posta	879	0.306	6.619	11-Apr-12	(+)	Hydeman *et al.* 2013
Hyperolius molleri	CAS 251580	1.5	STST	Bem Posta	879	0.306	6.619	11-Apr-12	(+)	Hydeman *et al.* 2013
Hyperolius molleri	CAS 251581	1.5	STST	Bem Posta	879	0.306	6.619	11-Apr-12	(+)	Hydeman *et al.* 2013
Hyperolius molleri	CAS 251582	1.5	STST	Bem Posta	879	0.306	6.619	11-Apr-12		Hydeman *et al.* 2013
Hyperolius molleri	CAS 251583	1.5	STST	Bem Posta	848	0.308	6.617	11-Apr-12	(+)	Hydeman *et al.* 2013
Hyperolius molleri	CAS 251584	1.5	STST	Bem Posta	848	0.308	6.617	11-Apr-12	(+)	Hydeman *et al.* 2013
Hyperolius molleri	CAS 251585	1.5	STST	Bem Posta	848	0.308	6.617	11-Apr-12		Hydeman *et al.* 2013
Hyperolius molleri	CAS 251586	1.5	STST	Bem Posta	848	0.308	6.617	11-Apr-12		Hydeman *et al.* 2013
Hyperolius molleri	CAS 251587	1.5	STST	Bem Posta	848	0.308	6.617	11-Apr-12	(+)	Hydeman *et al.* 2013
Hyperolius molleri	CAS 251588	1.5	STST	Bem Posta	848	0.308	6.617	11-Apr-12	(+)	Hydeman *et al.* 2013
Hyperolius molleri	CAS 251589	1.5	STST	Bem Posta	848	0.308	6.617	11-Apr-12		Hydeman *et al.* 2013
Hyperolius molleri	CAS 251590	1.5	STST	Bem Posta	848	0.308	6.617	11-Apr-12	(+)	Hydeman *et al.* 2013
Hyperolius molleri	CAS 251593	1.5	STST	Bom Sucesso	1156	0.288	6.613	12-Apr-12		Hydeman *et al.* 2013
Hyperolius molleri	CAS 251594	1.5	STST	Bom Sucesso	1156	0.288	6.613	12-Apr-12	(+)	Hydeman *et al.* 2013
Hyperolius molleri	CAS 251595	1.5	STST	Bom Sucesso	1156	0.288	6.613	12-Apr-12	(+)	Hydeman *et al.* 2013
Hyperolius molleri	CAS 251596	1.5	STST	Bom Sucesso	1156	0.288	6.613	12-Apr-12		Hydeman *et al.* 2013
Hyperolius molleri	CAS 251597	1.5	STST	Bom Sucesso	1159	0.290	6.612	12-Apr-12		Hydeman *et al.* 2013
Hyperolius molleri	CAS 251598	1.5	STST	Bom Sucesso	1159	0.290	6.612	12-Apr-12		Hydeman *et al.* 2013
Hyperolius molleri	CAS 251599	1.5	STST	Bom Sucesso	1159	0.290	6.612	12-Apr-12		Hydeman *et al.* 2013
Hyperolius molleri	CAS 251600	1.5	STST	Terra Batata	1020	0.288	6.624	12-Apr-12	(+)	Hydeman *et al.* 2013
Hyperolius molleri	CAS 251601	1.5	STST	Terra Batata	1020	0.288	6.624	12-Apr-12		Hydeman *et al.* 2013
Hyperolius molleri	CAS 251602	1.5	STST	Terra Batata	1020	0.288	6.624	12-Apr-12	(+)	Hydeman *et al.* 2013
Hyperolius molleri	CAS 251603	1.5	STST	Terra Batata	1020	0.288	6.624	12-Apr-12		Hydeman *et al.* 2013
Hyperolius molleri	CAS 251604	1.5	STST	Terra Batata	1020	0.288	6.624	12-Apr-12		Hydeman *et al.* 2013
Hyperolius molleri	CAS 251605	1.5	STST	Terra Batata	1020	0.288	6.624	12-Apr-12		Hydeman *et al.* 2013
Hyperolius molleri	CAS 251606	1.5	STST	Monte Café	758	0.296	6.638	12-Apr-12	(+)	Hydeman *et al.* 2013
Hyperolius molleri	CAS 251607	1.5	STST	Monte Café	758	0.296	6.638	12-Apr-12		Hydeman *et al.* 2013
Hyperolius molleri	CAS 251608	1.5	STST	Monte Café	758	0.296	6.638	12-Apr-12		Hydeman *et al.* 2013
Hyperolius molleri	CAS 251609	1.5	STST	Monte Café	758	0.296	6.638	12-Apr-12		Hydeman *et al.* 2013
Hyperolius molleri	CAS 251610	1.5	STST	Monte Café	758	0.296	6.638	12-Apr-12		Hydeman *et al.* 2013
Hyperolius molleri	CAS 251613	1.5	STST	Lagoa Amélia	1424	0.281	6.591	15-Apr-12	(+)	Hydeman *et al.* 2013
Hyperolius molleri	CAS 251614	1.5	STST	Lagoa Amélia	1424	0.281	6.591	15-Apr-12		Hydeman *et al.* 2013
Hyperolius molleri	CAS 251615	1.5	STST	Lagoa Amélia	1424	0.281	6.591	15-Apr-12	(+)	Hydeman *et al.* 2013
Hyperolius molleri	CAS 251616	1.5	STST	Lagoa Amélia	1424	0.281	6.591	15-Apr-12		Hydeman *et al.* 2013
Hyperolius molleri	CAS 251617	1.5	STST	Lagoa Amélia	1424	0.281	6.591	15-Apr-12		Hydeman *et al.* 2013
Hyperolius molleri	CAS 251618	1.5	STST	Lagoa Amélia	1424	0.281	6.591	15-Apr-12		Hydeman *et al.* 2013
Hyperolius molleri	CAS 251619	1.5	STST	Lagoa Amélia	1424	0.281	6.591	15-Apr-12	(+)	Hydeman *et al.* 2013
Hyperolius molleri	CAS 251622	1.5	STST	Caxueira	65	0.302	6.732	16-Apr-12		Hydeman *et al.* 2013
Hyperolius molleri	CAS 251623	1.5	STST	Caxueira	65	0.302	6.732	16-Apr-12		Hydeman *et al.* 2013
Hyperolius molleri	CAS 251624	1.5	STST	Caxueira	65	0.302	6.732	16-Apr-12		Hydeman *et al.* 2013
Hyperolius molleri	CAS 251625	1.5	STST	Caxueira	65	0.302	6.732	16-Apr-12		Hydeman *et al.* 2013
Hyperolius molleri	CAS 251626	1.5	STST	Caxueira	65	0.302	6.732	16-Apr-12		Hydeman *et al.* 2013
Hyperolius molleri	CAS 251627	1.5	STST	Caxueira	65	0.302	6.732	16-Apr-12	(+)	Hydeman *et al.* 2013
Hyperolius molleri	CAS 251628	1.5	STST	Caxueira	65	0.302	6.732	16-Apr-12		Hydeman *et al.* 2013
Hyperolius molleri	CAS 251629	1.5	STST	Caxueira	65	0.302	6.732	16-Apr-12		Hydeman *et al.* 2013
Hyperolius molleri	CAS 251630	1.5	STST	Caxueira	65	0.302	6.732	16-Apr-12		Hydeman *et al.* 2013
Hyperolius molleri	CAS 251631	1.5	STST	Caxueira	65	0.302	6.732	16-Apr-12	(+)	Hydeman *et al.* 2013
Hyperolius molleri	CAS 251632	1.5	STST	Caxueira	65	0.302	6.732	16-Apr-12		Hydeman *et al.* 2013
Hyperolius molleri	CAS 251633	1.5	STST	Caxueira	65	0.302	6.732	16-Apr-12		Hydeman *et al.* 2013
Hyperolius molleri	CAS 251634	1.5	STST	Caxueira	65	0.302	6.732	16-Apr-12		Hydeman *et al.* 2013
Hyperolius thomensis	CAS 233470	1.5	STST	Bom Sucesso	1323	0.276	6.606	7-May-06		Museum Specimen
Hyperolius thomensis	CAS 233471	1.5	STST	Bom Sucesso	1323	0.276	6.606	7-May-06	(+)	Museum Specimen
Hyperolius thomensis	CAS 233472	1.5	STST	Bom Sucesso	1323	0.276	6.606	7-May-06		Museum Specimen
Hyperolius thomensis	CAS 233473	1.5	STST	Bom Sucesso	1323	0.276	6.606	7-May-06		Museum Specimen
Hyperolius thomensis	CAS 233474	1.5	STST	Bom Sucesso	1323	0.276	6.606	7-May-06	(+)	Museum Specimen
Hyperolius thomensis	CAS 233475	1.5	STST	Bom Sucesso	1323	0.276	6.606	7-May-06		Museum Specimen
Hyperolius thomensis	CAS 233476	1.5	STST	Bom Sucesso	1323	0.276	6.606	7-May-06		Museum Specimen
Hyperolius thomensis	CAS 251635	1.5	STST	Bom Sucesso	1326	0.276	6.604	17-Apr-12		Hydeman *et al.* 2013

Species	Cat. No.	Aq. Index	Country	Locality	Elevation (m)	Lat	Long	Collected	*Bd*	Collection Method
Hyperolius thomensis	CAS 251636	1.5	STST	Bom Sucesso	1326	0.276	6.604	17-Apr-12	(+)	Hydeman *et al.* 2013
Hyperolius thomensis	CAS 251637	1.5	STST	Bom Sucesso	1326	0.276	6.604	17-Apr-12		Hydeman *et al.* 2013
Phrynobatrachus leveleve	CAS 218918	1.5	STST	Rio Contador	624	0.302	6.552	8-Apr-01		Museum Specimen
Phrynobatrachus leveleve	CAS 218919	1.5	STST	Rio Contador	624	0.302	6.552	8-Apr-01	(+)	Museum Specimen
Phrynobatrachus leveleve	CAS 218995	1.5	STST	Java	592	0.261	6.651	2-Apr-01		Museum Specimen
Phrynobatrachus leveleve	CAS 218996	1.5	STST	Java	592	0.261	6.651	2-Apr-01		Museum Specimen
Phrynobatrachus leveleve	CAS 218997	1.5	STST	Java	592	0.261	6.651	2-Apr-01		Museum Specimen
Phrynobatrachus leveleve	CAS 218999	1.5	STST	Java	592	0.261	6.651	2-Apr-01		Museum Specimen
Phrynobatrachus leveleve	CAS 219000	1.5	STST	Java	592	0.261	6.651	2-Apr-01		Museum Specimen
Phrynobatrachus leveleve	CAS 219001	1.5	STST	Java	592	0.261	6.651	2-Apr-01		Museum Specimen
Phrynobatrachus leveleve	CAS 219002	1.5	STST	Java	592	0.261	6.651	2-Apr-01		Museum Specimen
Phrynobatrachus leveleve	CAS 219003	1.5	STST	Java	592	0.261	6.651	2-Apr-01		Museum Specimen
Phrynobatrachus leveleve	CAS 219004	1.5	STST	Java	592	0.261	6.651	11-Apr-01		Museum Specimen
Phrynobatrachus leveleve	CAS 219027	1.5	STST	Caxueira	49	0.301	6.732	12-Apr-01		Museum Specimen
Phrynobatrachus leveleve	CAS 219051	1.5	STST	Bom Sucesso	1254	0.288	6.603	14-Apr-01		Museum Specimen
Phrynobatrachus leveleve	CAS 219052	1.5	STST	Bom Sucesso	1254	0.288	6.603	14-Apr-01		Museum Specimen
Phrynobatrachus leveleve	CAS 219053	1.5	STST	Bom Sucesso	1254	0.288	6.603	14-Apr-01		Museum Specimen
Phrynobatrachus leveleve	CAS 219064	1.5	STST	Rio Contador	653	0.307	6.551	15-Apr-01		Museum Specimen
Phrynobatrachus leveleve	CAS 219065	1.5	STST	Rio Contador	653	0.307	6.551	15-Apr-01		Museum Specimen
Phrynobatrachus leveleve	CAS 219066	1.5	STST	Rio Contador	653	0.307	6.551	15-Apr-01		Museum Specimen
Phrynobatrachus leveleve	CAS 219067	1.5	STST	Rio Contador	653	0.307	6.551	15-Apr-01		Museum Specimen
Phrynobatrachus leveleve	CAS 219264	1.5	STST	Água Grande	11	0.333	6.729	25-Apr-01		Museum Specimen
Phrynobatrachus leveleve	CAS 219265	1.5	STST	Água Grande	11	0.333	6.729	25-Apr-01	(+)	Museum Specimen
Phrynobatrachus leveleve	CAS 219266	1.5	STST	Água Grande	11	0.333	6.729	25-Apr-01		Museum Specimen
Phrynobatrachus leveleve	CAS 219267	1.5	STST	Água Grande	11	0.333	6.729	25-Apr-01		Museum Specimen
Phrynobatrachus leveleve	CAS 219268	1.5	STST	Água Grande	11	0.333	6.729	25-Apr-01		Museum Specimen
Phrynobatrachus leveleve	CAS 219319	1.5	STST	Bom Sucesso	1221	0.285	6.606	5-May-01		Museum Specimen
Phrynobatrachus leveleve	CAS 219320	1.5	STST	Bom Sucesso	1221	0.285	6.606	5-May-01		Museum Specimen
Phrynobatrachus leveleve	CAS 219321	1.5	STST	Bom Sucesso	1221	0.285	6.606	5-May-01		Museum Specimen
Phrynobatrachus leveleve	CAS 219406	1.5	STST	Java	592	0.261	6.651	2-Apr-01		Museum Specimen
Phrynobatrachus leveleve	CAS 219407	1.5	STST	Java	592	0.261	6.651	2-Apr-01		Museum Specimen
Phrynobatrachus leveleve	CAS 219408	1.5	STST	Java	592	0.261	6.651	2-Apr-01		Museum Specimen
Phrynobatrachus leveleve	CAS 219409	1.5	STST	Java	592	0.261	6.651	2-Apr-01		Museum Specimen
Phrynobatrachus leveleve	CAS 233677	1.5	STST	Abade	400	0.254	6.645	19-May-06		Museum Specimen
Phrynobatrachus leveleve	CAS 233678	1.5	STST	Abade	400	0.254	6.645	19-May-06		Museum Specimen
Phrynobatrachus leveleve	CAS 233679	1.5	STST	Abade	400	0.254	6.645	19-May-06		Museum Specimen
Phrynobatrachus leveleve	CAS 233680	1.5	STST	Abade	400	0.254	6.645	19-May-06	(+)	Museum Specimen
Phrynobatrachus leveleve	CAS 233681	1.5	STST	Abade	400	0.254	6.645	19-May-06		Museum Specimen
Phrynobatrachus leveleve	CAS 233682	1.5	STST	Abade	400	0.254	6.645	19-May-06	(+)	Museum Specimen
Phrynobatrachus leveleve	CAS 233683	1.5	STST	Abade	400	0.254	6.645	19-May-06		Museum Specimen
Phrynobatrachus leveleve	CAS 233684	1.5	STST	Abade	400	0.254	6.645	19-May-06		Museum Specimen
Phrynobatrachus leveleve	CAS 233685	1.5	STST	Abade	400	0.254	6.645	19-May-06	(+)	Museum Specimen
Phrynobatrachus leveleve	CAS 233686	1.5	STST	Abade	400	0.254	6.645	19-May-06		Museum Specimen
Phrynobatrachus leveleve	CAS 233687	1.5	STST	Abade	400	0.254	6.645	19-May-06	(+)	Museum Specimen
Phrynobatrachus leveleve	CAS 233688	1.5	STST	Abade	400	0.254	6.645	19-May-06		Museum Specimen
Phrynobatrachus leveleve	CAS 233689	1.5	STST	Abade	400	0.254	6.645	19-May-06		Museum Specimen
Phrynobatrachus leveleve	CAS 233690	1.5	STST	Abade	400	0.254	6.645	19-May-06		Museum Specimen
Phrynobatrachus leveleve	CAS 233691	1.5	STST	Abade	400	0.254	6.645	19-May-06	(+)	Museum Specimen
Phrynobatrachus leveleve	CAS 233698	1.5	STST	Abade	400	0.254	6.645	20-May-06		Museum Specimen
Phrynobatrachus leveleve	CAS 233699	1.5	STST	Abade	400	0.254	6.645	20-May-06		Museum Specimen
Phrynobatrachus leveleve	CAS 233700	1.5	STST	Lagoa Amélia	1444	0.282	6.591	21-May-06		Museum Specimen
Phrynobatrachus leveleve	CAS 233701	1.5	STST	Lagoa Amélia	1444	0.282	6.591	21-May-06		Museum Specimen
Phrynobatrachus leveleve	CAS 233704	1.5	STST	Abade	400	0.254	6.645	26-May-06		Museum Specimen
Phrynobatrachus leveleve	CAS 251591	1.5	STST	Bem Posta	840	0.309	6.618	11-Apr-12		Hydeman *et al.* 2013
Phrynobatrachus leveleve	CAS 251592	1.5	STST	Bem Posta	840	0.309	6.618	11-Apr-12		Hydeman *et al.* 2013
Phrynobatrachus leveleve	CAS 252822	1.5	STST	Rio Contador	644	0.316	6.549	3-Nov-11	(+)	Museum Specimen
Phrynobatrachus leveleve	CAS 252823	1.5	STST	Rio Contador	644	0.316	6.549	3-Nov-11		Museum Specimen
Ptychadena newtoni	CAS 219250	2	STST	Água Grande	11	0.333	6.729	25-Apr-01		Museum Specimen
Ptychadena newtoni	CAS 219251	2	STST	Água Grande	11	0.333	6.729	25-Apr-01		Museum Specimen
Ptychadena newtoni	CAS 219252	2	STST	Água Grande	11	0.333	6.729	25-Apr-01		Museum Specimen
Ptychadena newtoni	CAS 219253	2	STST	Água Grande	11	0.333	6.729	25-Apr-01		Museum Specimen
Ptychadena newtoni	CAS 219254	2	STST	Água Grande	11	0.333	6.729	25-Apr-01		Museum Specimen
Ptychadena newtoni	CAS 219255	2	STST	Água Grande	11	0.333	6.729	25-Apr-01		Museum Specimen
Ptychadena newtoni	CAS 219256	2	STST	Água Grande	11	0.333	6.729	25-Apr-01		Museum Specimen
Ptychadena newtoni	CAS 219257	2	STST	Água Grande	11	0.333	6.729	25-Apr-01		Museum Specimen
Ptychadena newtoni	CAS 219258	2	STST	Água Grande	11	0.333	6.729	25-Apr-01		Museum Specimen
Ptychadena newtoni	CAS 219259	2	STST	Água Grande	11	0.333	6.729	25-Apr-01		Museum Specimen
Ptychadena newtoni	CAS 219260	2	STST	Água Grande	11	0.333	6.729	25-Apr-01		Museum Specimen
Ptychadena newtoni	CAS 219261	2	STST	Água Grande	11	0.333	6.729	25-Apr-01		Museum Specimen
Ptychadena newtoni	CAS 219262	2	STST	Água Grande	11	0.333	6.729	25-Apr-01	(+)	Museum Specimen
Ptychadena newtoni	CAS 219313	2	STST	Água Grande	11	0.333	6.729	4-May-01		Museum Specimen
Ptychadena newtoni	CAS 219314	2	STST	Água Grande	11	0.333	6.729	4-May-01		Museum Specimen
Ptychadena newtoni	CAS 219315	2	STST	Água Grande	11	0.333	6.729	4-May-01		Museum Specimen
Ptychadena newtoni	CAS 219316	2	STST	Água Grande	11	0.333	6.729	4-May-01		Museum Specimen
Ptychadena newtoni	CAS 219317	2	STST	Água Grande	11	0.333	6.729	4-May-01		Museum Specimen
Ptychadena newtoni	CAS 251620	2	STST	Caxueira	65	0.302	6.732	16-Apr-12		Hydeman *et al.* 2013
Ptychadena newtoni	CAS 251621	2	STST	Caxueira	65	0.302	6.732	16-Apr-12	(+)	Hydeman *et al.* 2013
Ptychadena newtoni	CAS 252829	2	STST	Santo Amaro	54	0.368	6.686	3-Nov-11		Museum Specimen
Schistometopum thomense	CAS 19023	1	STST	unknown	unknown	N/A	N/A	1935-1936		Museum Specimen
Schistometopum thomense	CAS 84205	1	STST	Bom Sucesso	unknown	N/A	N/A	14-Aug-49		Museum Specimen
Schistometopum thomense	CAS 218734	1	STST	Java	592	0.261	6.651	31-Mar-01		Museum Specimen

Species	Cat. No.	Aq. Index	Country	Locality	Elevation (m)	Lat	Long	Collected	*Bd*	Collection Method
Schistometopum thomense	CAS 218735	1	STST	Java	592	0.261	6.651	31-Mar-01	(+)	Museum Specimen
Schistometopum thomense	CAS 218736	1	STST	Java	592	0.261	6.651	31-Mar-01	(+)	Museum Specimen
Schistometopum thomense	CAS 218737	1	STST	Java	592	0.261	6.651	31-Mar-01	(+)	Museum Specimen
Schistometopum thomense	CAS 218738	1	STST	Java	592	0.261	6.651	31-Mar-01	(+)	Museum Specimen
Schistometopum thomense	CAS 218739	1	STST	Java	592	0.261	6.651	31-Mar-01	(+)	Museum Specimen
Schistometopum thomense	CAS 218740	1	STST	Java	592	0.261	6.651	31-Mar-01	(+)	Museum Specimen
Schistometopum thomense	CAS 218741	1	STST	Java	592	0.261	6.651	31-Mar-01	(+)	Museum Specimen
Schistometopum thomense	CAS 218742	1	STST	Java	592	0.261	6.651	31-Mar-01	(+)	Museum Specimen
Schistometopum thomense	CAS 218743	1	STST	Java	592	0.261	6.651	31-Mar-01	(+)	Museum Specimen
Schistometopum thomense	CAS 218744	1	STST	Java	592	0.261	6.651	31-Mar-01	(+)	Museum Specimen
Schistometopum thomense	CAS 218745	1	STST	Java	592	0.261	6.651	31-Mar-01	(+)	Museum Specimen
Schistometopum thomense	CAS 218746	1	STST	Java	592	0.261	6.651	31-Mar-01		Museum Specimen
Schistometopum thomense	CAS 218747	1	STST	Java	592	0.261	6.651	31-Mar-01		Museum Specimen
Schistometopum thomense	CAS 218748	1	STST	Java	592	0.261	6.651	31-Mar-01		Museum Specimen
Schistometopum thomense	CAS 218749	1	STST	Java	592	0.261	6.651	31-Mar-01		Museum Specimen
Schistometopum thomense	CAS 218773	1	STST	Between Santa Luzia and Java	unknown	N/A	N/A	2-Apr-01		Museum Specimen
Schistometopum thomense	CAS 218774	1	STST	Milagrosa	449	0.275	6.653	2-Apr-01	(+)	Museum Specimen
Schistometopum thomense	CAS 218775	1	STST	Milagrosa	449	0.275	6.653	2-Apr-01	(+)	Museum Specimen
Schistometopum thomense	CAS 218776	1	STST	Milagrosa	449	0.275	6.653	2-Apr-01	(+)	Museum Specimen
Schistometopum thomense	CAS 218777	1	STST	Milagrosa	449	0.275	6.653	2-Apr-01		Museum Specimen
Schistometopum thomense	CAS 218778	1	STST	Milagrosa	449	0.275	6.653	2-Apr-01		Museum Specimen
Schistometopum thomense	CAS 218779	1	STST	Milagrosa	449	0.275	6.653	2-Apr-01		Museum Specimen
Schistometopum thomense	CAS 218780	1	STST	Milagrosa	449	0.275	6.653	2-Apr-01		Museum Specimen
Schistometopum thomense	CAS 218781	1	STST	Milagrosa	449	0.275	6.653	2-Apr-01		Museum Specimen
Schistometopum thomense	CAS 218782	1	STST	Milagrosa	449	0.275	6.653	2-Apr-01		Museum Specimen
Schistometopum thomense	CAS 218783	1	STST	Milagrosa	449	0.275	6.653	2-Apr-01	(+)	Museum Specimen
Schistometopum thomense	CAS 218902	1	STST	Abade	688	0.258	6.614	6-Apr-01		Museum Specimen
Schistometopum thomense	CAS 218903	1	STST	Abade	513	0.254	6.630	6-Apr-01		Museum Specimen
Schistometopum thomense	CAS 218904	1	STST	Abade	513	0.254	6.630	6-Apr-01		Museum Specimen
Schistometopum thomense	CAS 218905	1	STST	Abade	513	0.254	6.630	6-Apr-01		Museum Specimen
Schistometopum thomense	CAS 218914	1	STST	Rio Contador	452	0.310	6.552	8-Apr-01		Museum Specimen
Schistometopum thomense	CAS 218915	1	STST	Rio Contador	452	0.310	6.552	8-Apr-01		Museum Specimen
Schistometopum thomense	CAS 218916	1	STST	Rio Contador	624	0.302	6.552	8-Apr-01		Museum Specimen
Schistometopum thomense	CAS 218917	1	STST	Rio Contador	653	0.306	6.551	8-Apr-01		Museum Specimen
Schistometopum thomense	CAS 218921	1	STST	Rio Contador	658	0.312	6.550	8-Apr-01		Museum Specimen
Schistometopum thomense	CAS 218923	1	STST	Rio Contador	599	0.316	6.551	8-Apr-01		Museum Specimen
Schistometopum thomense	CAS 218960	1	STST	Canavial	148	0.381	6.649	11-Apr-01		Museum Specimen
Schistometopum thomense	CAS 218961	1	STST	Canavial	148	0.381	6.649	11-Apr-01		Museum Specimen
Schistometopum thomense	CAS 219031	1	STST	Rio do Ouro	169	0.365	6.645	13-Apr-01		Museum Specimen
Schistometopum thomense	CAS 219032	1	STST	Rio do Ouro	169	0.365	6.645	13-Apr-01		Museum Specimen
Schistometopum thomense	CAS 219033	1	STST	Rio do Ouro	169	0.365	6.645	13-Apr-01		Museum Specimen
Schistometopum thomense	CAS 219034	1	STST	Rio do Ouro	169	0.365	6.645	13-Apr-01		Museum Specimen
Schistometopum thomense	CAS 219074	1	STST	Canavial	99	0.384	6.654	18-Apr-01		Museum Specimen
Schistometopum thomense	CAS 219075	1	STST	Canavial	99	0.384	6.654	18-Apr-01		Museum Specimen
Schistometopum thomense	CAS 219286	1	STST	Caxueira	49	0.301	6.732	30-Apr-01		Museum Specimen
Schistometopum thomense	CAS 219292	1	STST	Roça Sao João	146	0.142	6.645	2-May-01		Museum Specimen
Schistometopum thomense	CAS 219295	1	STST	Cruzeiro	338	0.286	6.678	4-May-01		Museum Specimen
Schistometopum thomense	CAS 219296	1	STST	Cruzeiro	338	0.286	6.678	4-May-01		Museum Specimen
Schistometopum thomense	CAS 219297	1	STST	Cruzeiro	338	0.286	6.678	4-May-01		Museum Specimen
Schistometopum thomense	CAS 219298	1	STST	Cruzeiro	338	0.286	6.678	4-May-01		Museum Specimen
Schistometopum thomense	CAS 219299	1	STST	Cruzeiro	338	0.286	6.678	4-May-01		Museum Specimen
Schistometopum thomense	CAS 219300	1	STST	Cruzeiro	338	0.286	6.678	4-May-01		Museum Specimen
Schistometopum thomense	CAS 219301	1	STST	Cruzeiro	338	0.286	6.678	4-May-01		Museum Specimen
Schistometopum thomense	CAS 219302	1	STST	Cruzeiro	338	0.286	6.678	4-May-01		Museum Specimen
Schistometopum thomense	CAS 219303	1	STST	Cruzeiro	338	0.286	6.678	4-May-01		Museum Specimen
Schistometopum thomense	CAS 219304	1	STST	Cruzeiro	338	0.286	6.678	4-May-01		Museum Specimen
Schistometopum thomense	CAS 219307	1	STST	Cruzeiro	338	0.286	6.678	4-May-01		Museum Specimen
Schistometopum thomense	CAS 219308	1	STST	Cruzeiro	338	0.286	6.678	4-May-01		Museum Specimen
Schistometopum thomense	CAS 219309	1	STST	Cruzeiro	338	0.286	6.678	4-May-01		Museum Specimen
Schistometopum thomense	CAS 219312	1	STST	Cruzeiro	302	0.288	6.681	5-May-01		Museum Specimen
Schistometopum thomense	CAS 233451	1	STST	Rio Contador	600	unknown	unknown	30-Apr-06		Museum Specimen
Schistometopum thomense	CAS 233452	1	STST	Rio Contador	600	unknown	unknown	30-Apr-06		Museum Specimen
Schistometopum thomense	CAS 233453	1	STST	Milagrosa	500	0.276	6.650	3-May-06		Museum Specimen
Schistometopum thomense	CAS 233454	1	STST	Milagrosa	500	0.276	6.650	3-May-06		Museum Specimen
Schistometopum thomense	CAS 233455	1	STST	Milagrosa	500	0.276	6.650	3-May-06		Museum Specimen
Schistometopum thomense	CAS 233456	1	STST	Milagrosa	500	0.276	6.650	3-May-06		Museum Specimen
Schistometopum thomense	CAS 233457	1	STST	Milagrosa	500	0.276	6.650	3-May-06		Museum Specimen
Schistometopum thomense	CAS 233458	1	STST	Milagrosa	500	0.276	6.650	3-May-06	(+)	Museum Specimen
Schistometopum thomense	CAS 233459	1	STST	Milagrosa	500	0.276	6.650	3-May-06		Museum Specimen
Schistometopum thomense	CAS 233460	1	STST	Milagrosa	500	0.276	6.650	3-May-06		Museum Specimen
Schistometopum thomense	CAS 233485	1	STST	Rio Maria Luisa	28	0.328	6.512	8-May-06		Museum Specimen
Schistometopum thomense	CAS 233486	1	STST	Rio Maria Luisa	28	0.328	6.512	8-May-06		Museum Specimen
Schistometopum thomense	CAS 233487	1	STST	Rio Maria Luisa	28	0.328	6.512	8-May-06		Museum Specimen
Schistometopum thomense	CAS 233571	1	STST	Rio Lemba bridge	15	0.247	6.466	15-May-06		Museum Specimen
Schistometopum thomense	CAS 233572	1	STST	Rio Lemba bridge	15	0.247	6.466	15-May-06		Museum Specimen
Schistometopum thomense	CAS 233573	1	STST	Rio Lemba bridge	15	0.247	6.466	15-May-06		Museum Specimen
Schistometopum thomense	CAS 233574	1	STST	Rio Lemba bridge	15	0.247	6.466	15-May-06	(+)	Museum Specimen
Schistometopum thomense	CAS 233575	1	STST	Rio Lemba bridge	15	0.247	6.466	15-May-06		Museum Specimen
Schistometopum thomense	CAS 233576	1	STST	Rio Lemba bridge	15	0.247	6.466	15-May-06		Museum Specimen
Schistometopum thomense	CAS 233577	1	STST	Porto Alegre	18	0.036	6.531	17-May-06		Museum Specimen
Schistometopum thomense	CAS 233578	1	STST	Porto Alegre	18	0.036	6.531	17-May-06		Museum Specimen

Species	Cat. No.	Aq. Index	Country	Locality	Elevation (m)	Lat	Long	Collected	*Bd*	Collection Method
Schistometopum thomense	CAS 233579	1	STST	Porto Alegre	18	0.036	6.531	17-May-06		Museum Specimen
Schistometopum thomense	CAS 233580	1	STST	Porto Alegre	18	0.036	6.531	17-May-06		Museum Specimen
Schistometopum thomense	CAS 233581	1	STST	Porto Alegre	18	0.036	6.531	17-May-06		Museum Specimen
Schistometopum thomense	CAS 233582	1	STST	Porto Alegre	18	0.036	6.531	17-May-06		Museum Specimen
Schistometopum thomense	CAS 233583	1	STST	Porto Alegre	18	0.036	6.531	17-May-06		Museum Specimen
Schistometopum thomense	CAS 233584	1	STST	Porto Alegre	18	0.036	6.531	17-May-06		Museum Specimen
Schistometopum thomense	CAS 233585	1	STST	Porto Alegre	18	0.036	6.531	17-May-06		Museum Specimen
Schistometopum thomense	CAS 233586	1	STST	Porto Alegre	18	0.036	6.531	17-May-06		Museum Specimen
Schistometopum thomense	CAS 233587	1	STST	Porto Alegre	18	0.036	6.531	17-May-06		Museum Specimen
Schistometopum thomense	CAS 233588	1	STST	Porto Alegre	18	0.036	6.531	17-May-06		Museum Specimen
Schistometopum thomense	CAS 233589	1	STST	Porto Alegre	18	0.036	6.531	17-May-06		Museum Specimen
Schistometopum thomense	CAS 233590	1	STST	Porto Alegre	18	0.036	6.531	17-May-06		Museum Specimen
Schistometopum thomense	CAS 233591	1	STST	Porto Alegre	18	0.036	6.531	17-May-06		Museum Specimen
Schistometopum thomense	CAS 233592	1	STST	Porto Alegre	18	0.036	6.531	17-May-06		Museum Specimen
Schistometopum thomense	CAS 233593	1	STST	Porto Alegre	18	0.036	6.531	17-May-06		Museum Specimen
Schistometopum thomense	CAS 233594	1	STST	Porto Alegre	18	0.036	6.531	17-May-06		Museum Specimen
Schistometopum thomense	CAS 233595	1	STST	Porto Alegre	18	0.036	6.531	17-May-06		Museum Specimen
Schistometopum thomense	CAS 233596	1	STST	Porto Alegre	18	0.036	6.531	17-May-06		Museum Specimen
Schistometopum thomense	CAS 233619	1	STST	Java	600	0.262	6.651	18-May-06		Museum Specimen
Schistometopum thomense	CAS 233620	1	STST	Java	600	0.262	6.651	18-May-06		Museum Specimen
Schistometopum thomense	CAS 233621	1	STST	Abade	410	0.255	6.650	18-May-06		Museum Specimen
Schistometopum thomense	CAS 233622	1	STST	Abade	410	0.255	6.650	18-May-06		Museum Specimen
Schistometopum thomense	CAS 233623	1	STST	Abade	410	0.255	6.650	18-May-06		Museum Specimen
Schistometopum thomense	CAS 233624	1	STST	Abade	410	0.255	6.650	18-May-06		Museum Specimen
Schistometopum thomense	CAS 233625	1	STST	Abade	410	0.255	6.650	18-May-06		Museum Specimen
Schistometopum thomense	CAS 233626	1	STST	Abade	410	0.255	6.650	18-May-06		Museum Specimen
Schistometopum thomense	CAS 233629	1	STST	Abade	410	0.255	6.650	18-May-06		Museum Specimen
Schistometopum thomense	CAS 233630	1	STST	Abade	410	0.255	6.650	18-May-06		Museum Specimen
Schistometopum thomense	CAS 233635	1	STST	Abade	410	0.255	6.650	18-May-06		Museum Specimen
Schistometopum thomense	CAS 233641	1	STST	Abade	410	0.255	6.650	18-May-06		Museum Specimen
Schistometopum thomense	CAS 233663	1	STST	Abade	400	0.254	6.645	18-May-06		Museum Specimen
Schistometopum thomense	CAS 233664	1	STST	Abade	400	0.254	6.645	18-May-06		Museum Specimen
Schistometopum thomense	CAS 233665	1	STST	Abade	400	0.254	6.645	18-May-06		Museum Specimen
Schistometopum thomense	CAS 233666	1	STST	Abade	400	0.254	6.645	18-May-06		Museum Specimen
Schistometopum thomense	CAS 233667	1	STST	Abade	400	0.254	6.645	18-May-06		Museum Specimen
Schistometopum thomense	CAS 233668	1	STST	Abade	400	0.254	6.645	18-May-06		Museum Specimen
Schistometopum thomense	CAS 233669	1	STST	Abade	400	0.254	6.645	18-May-06		Museum Specimen
Schistometopum thomense	CAS 233670	1	STST	Abade	400	0.254	6.645	18-May-06		Museum Specimen
Schistometopum thomense	CAS 233671	1	STST	Abade	400	0.254	6.645	18-May-06		Museum Specimen
Schistometopum thomense	CAS 233672	1	STST	Abade	400	0.254	6.645	18-May-06		Museum Specimen
Schistometopum thomense	CAS 251565	1	STST	Abade	518	0.254	6.630	5-Apr-12		Museum Specimen
Schistometopum thomense	CAS 251566	1	STST	Abade	518	0.254	6.630	5-Apr-12		Museum Specimen
Schistometopum thomense	CAS 251567	1	STST	Abade	518	0.254	6.630	5-Apr-12		Museum Specimen
Schistometopum thomense	CAS 251568	1	STST	Abade	518	0.254	6.630	5-Apr-12	(+)	Museum Specimen
Schistometopum thomense	CAS 251569	1	STST	Abade	518	0.254	6.630	5-Apr-12		Museum Specimen
Schistometopum thomense	CAS 251570	1	STST	Abade	518	0.254	6.630	5-Apr-12		Museum Specimen
Schistometopum thomense	CAS 251574	1	STST	Vale do Contador	616	0.286	6.570	9-Apr-12	(+)	Hydeman *et al.* 2013
Schistometopum thomense	CAS 251575	1	STST	Vale do Contador	616	0.286	6.570	9-Apr-12		Hydeman *et al.* 2013
Schistometopum thomense	CAS 251576	1	STST	Vale do Contador	616	0.286	6.570	9-Apr-12		Hydeman *et al.* 2013
Schistometopum thomense	CAS 251577	1	STST	Vale do Contador	616	0.286	6.570	9-Apr-12		Hydeman *et al.* 2013
Schistometopum thomense	CAS 251578	1	STST	Vale do Contador	522	0.292	6.567	9-Apr-12		Hydeman *et al.* 2013
Schistometopum thomense	CAS 252814	1	STST	Bom Sucesso	1311	0.285	6.602	31-Oct-11		Museum Specimen
Schistometopum thomense	CAS 252815	1	STST	Bom Sucesso	1311	0.285	6.602	31-Oct-11		Museum Specimen
Schistometopum thomense	CAS 252824	1	STST	Rio Contador	644	0.316	6.549	3-Nov-11		Museum Specimen
Schistometopum thomense	CAS 252825	1	STST	Rio Contador	644	0.316	6.549	3-Nov-11		Museum Specimen
Schistometopum thomense	CAS 252826	1	STST	Rio Contador	644	0.316	6.549	3-Nov-11		Museum Specimen
Schistometopum thomense	CAS 252827	1	STST	Rio Contador	644	0.316	6.549	3-Nov-11		Museum Specimen
Schistometopum thomense	CAS 252828	1	STST	Rio Contador	644	0.316	6.549	3-Nov-11		Museum Specimen
