# Supplementary material for: Prevalence and genetic diversity of Batrachochytrium dendrobatidis in Central African island and continental amphibian communities
Source: Ecol Evol. 2017 Aug 22;7(19):7729–38. doi: 10.1002/ece3.3309 (PMC5632636; doi:10.1002/ece3.3309)
Supplement: Supplementary file 2 [file ECE3-7-7729-s002.pdf]

Table S2: Sampling localities and voucher information for *Bd*-positive samples with ITS sequences. Abbreviations as follow: Equatorial Guinea - Bioko Island (EGBK), Gabon - Estuaire (GAES), Gabon - Ogooué-Ivindo (GAOI), Gabon - Woleu-Ntem (WAWN), São Tomé + Príncipe - São Tomé Island (STST), São Tomé + Príncipe - Príncipe Island (STPR), Cornell University Museum of Vertebrates (CU), the California Academy of Sciences (CAS), the North Carolina Museum of Natural Sciences (NCSM), and the Museum of Comparative Zoology (MCZ).

Species	Ca. No.	Country	Locality	Method	BZ01	BZ02	BZ03	BZ09	BZ12	BZ19	GOG01	PR01	PR02	ST01	ST02	ST03	ST04	ST05	GA01	GA02
Afrixalus fulvovittatus	CU 14936	GAOI	Ivindo National Park, Ipassa Station	PCR only	1															
Afrixalus paradorsalis	CU 14928	GAOI	Ivindo National Park, Ipassa Station	PCR only	1															
Arthroleptis sp.	MCZ A-139754	GAOI	Ivindo National Park, Ipassa Station	PCR only	1															
Arthroleptis sp.	MCZ A-139776	GAOI	Ivindo National Park, Ipassa Station	PCR only	1															
Arthroleptis sp.	NCSM 76805	GAOI	Ivindo National Park, Ipassa Station	PCR only	1															
Cardioglossa elegans	MCZ A-139646	GAES	Monts de Cristal National Park, Kinguélé	PCR only		1														
Cardioglossa leucomystax	NCSM 76808	GAWN	Monts de Cristal National Park, Tchimbélé	PCR only	1															
Chiromantis rufescens	MCZ A-139786	GAOI	Ivindo National Park, Ipassa Station	PCR only	1															
Chiromantis rufescens	MCZ A-139787	GAOI	Ivindo National Park, Ipassa Station	PCR only	1															
Chiromantis rufescens	MCZ A-139790	GAOI	Ivindo National Park, Ipassa Station	PCR only					1											
Chiromantis rufescens	MCZ A-139649	GAES	Monts de Cristal National Park, Kinguélé	PCR only	1															
Hylarana sp.	MCZ A-139717	GAOI	Ivindo National Park, Ipassa Station	PCR only	1															
Hylarana sp.	MCZ A-139746	GAOI	Ivindo National Park, Ipassa Station	PCR only	1															
Hylarana sp.	MCZ A-139773	GAOI	Ivindo National Park, Ipassa Station	PCR only	1															
Hylarana sp.	MCZ A-139815	GAWN	Monts de Cristal National Park, Tchimbélé	PCR only	1															
Hyperolius ocellatus	CU 14892	GAOI	Ivindo National Park, Ipassa Station	PCR only	1															
Hyperolius ocellatus	CU 14960	GAOI	Ivindo National Park, Ipassa Station	PCR only	1															
Hyperolius ocellatus	CU 14895	GAES	Monts de Cristal National Park, Kinguélé	PCR only	1															
Hyperolius tuberculatus	CU 14911	GAWN	Monts de Cristal National Park, Tchimbélé	PCR only	1															
Leptopelis aubryi	NCSM 76829	GAOI	Ivindo National Park, Ipassa Station	PCR only	1															
Leptopelis brevirostris	MCZ A-139795	GAOI	Ivindo National Park, Ipassa Station	PCR only	1															
Leptopelis crystallinoron	NCSM 76820	GAES	Monts de Cristal National Park, Kinguélé	PCR only	1															
Leptopelis crystallinoron	MCZ A-139811	GAWN	Monts de Cristal National Park, Tchimbélé	PCR only	1															
Leptopelis ocellatus	MCZ A-139735	GAOI	Ivindo National Park, Ipassa Station	PCR only					1											
Leptopelis ocellatus	MCZ A-139731	GAOI	Ivindo National Park, Ipassa Station	PCR only	1															
Leptopelis ocellatus	MCZ A-139729	GAOI	Ivindo National Park, Ipassa Station	PCR only					1											
Leptopelis ocellatus	NCSM 76824	GAOI	Ivindo National Park, Ipassa Station	PCR only			1													
Leptopelis sp.	MCZ A-139814	GAWN	Monts de Cristal National Park, Tchimbélé	PCR only	1															
Leptopelis sp.	MCZ A-139741	GAOI	Ivindo National Park, Ipassa Station	PCR only	1															
Leptopelis sp.	MCZ A-139799	GAOI	Ivindo National Park, Ipassa Station	PCR only	1															
Phrynobatrachus auritus	MCZ A-139639	GAES	Monts de Cristal National Park, Kinguélé	PCR only	1															
Phrynobatrachus auritus	MCZ A-139812	GAWN	Monts de Cristal National Park, Tchimbélé	PCR only	1															
Phrynobatrachus cornutus	CAS 207893	EGBK	Bioko, Río Iladyi	cloned	1						1									
Phrynobatrachus dispar	CAS 233550	STPR	Príncipe, Base camp, Pico de Príncipe	cloned		1														
Phrynobatrachus dispar	CAS 233556	STPR	Príncipe, Base camp, Pico de Príncipe	cloned	1							1	1							
Phrynobatrachus leveleve	CAS 233685	STST	São Tomé, Abade	cloned		1		1		1	1									
Ptychadena sp.	NCSM 78946	GAOI	Ivindo, Rougier Gabon Forestry Concession	cloned		1													1	1
Ptychadena sp.	MCZ A-139744	GAOI	Ivindo National Park, Ipassa Station	PCR only	1															
Schistometopum thomense	CAS 218738	STST	São Tomé, Java	cloned	1	1								1	1			1		
Schistometopum thomense	CAS 218741	STST	São Tomé, Java	cloned	1	1											1			
Schistometopum thomense	CAS 233574	STST	São Tomé, Río Lemba Bridge	cloned	1	1										1				
Scotobleps gabonicus	MCZ A-139706	GAES	Monts de Cristal National Park, Kinguélé	PCR only	1															
Scotobleps gabonicus	MCZ A-139818	GAWN	Monts de Cristal National Park, Tchimbélé	PCR only	1															
Trichobatrachus robustus	MCZ A-139819	GAWN	Monts de Cristal National Park, Tchimbélé	PCR only	1															
